# Simple two-step synthesis of 2,4-disubstituted pyrroles and 3,5-disubstituted pyrrole-2-carbonitriles from enones

**DOI:** 10.3762/bjoc.10.44

**Published:** 2014-02-24

**Authors:** Murat Kucukdisli, Dorota Ferenc, Marcel Heinz, Christine Wiebe, Till Opatz

**Affiliations:** 1 Institute of Organic Chemistry, Johannes Gutenberg University of Mainz, Duesbergweg 10–14, 55128 Mainz, Germany; 2 Department of Chemistry, University of Hamburg, Martin-Luther-King-Platz 6, 20146 Hamburg, Germany

**Keywords:** α-aminonitriles, cyclization, heterocycles, microwave-assisted synthesis, pyrroles

## Abstract

The cyclocondensation of enones with aminoacetonitrile furnishes 3,4-dihydro-2*H*-pyrrole-2-carbonitriles which can be readily converted to 2,4-disubstituted pyrroles by microwave-induced dehydrocyanation. Alternatively, oxidation of the intermediates produces 3,5-disubstituted pyrrole-2-carbonitriles.

## Introduction

Heterocycles are the largest class of organic compounds [1]. Among them, pyrroles have a distinguished position in the chemistry of living organisms due to their close biogenetic connection to the porphyrins, the chlorins, and the corrins. Furthermore, they are regarded as privileged structures by synthetic chemists because of widespread applications in medicinal chemistry [2–3] and materials science [4]. Not surprisingly, numerous synthetic methods [5–27] are still being developed for this compound class although many classical methods such as Hantzsch [28], Knorr [29], Paal–Knorr [30], and Barton–Zard [31] have already found their way into the textbooks of organic chemistry.

**Scheme 1:**
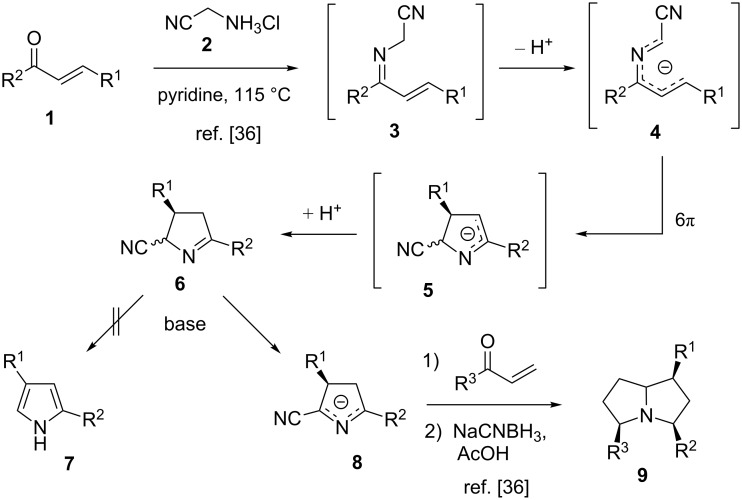
Synthesis and conversion of 3,4-dihydro-2*H*-pyrrole-2-carbonitriles **6**.

α-(Alkylideneamino)nitriles can be used as versatile and readily accessible pronucleophiles, e.g. for the construction of γ-amino acids [32], pyrrolidines [33], as well as pyrroles [34–35]. If an additional conjugated double bond is present, the anions of α-(alkylideneamino)nitriles **3** can undergo an electrocyclic ring closure to furnish 3,4-dihydro-2*H*-pyrrole-2-carbonitriles **6** after reprotonation [36]. If the products are devoid of an additional substituent in 2-position, the cyclocondensation can be simply effected by refluxing a mixture of the enone **1** and aminoacetonitrile hydrochloride (**2**) in pyridine. The base-induced dehydrocyanation of compounds **6** does not produce appreciable yields of pyrroles **7** since the abstraction of the most acidic proton leads to a stable anion reluctant to undergo α-elimination of cyanide. Instead, the anions **8** can be added in vinylogous fashion to α,β-unsaturated carbonyls producing intermediates, the exhaustive reduction of which leads to highly substituted pyrrolizidines **9** (Scheme 1).

## Results and Discussion

In view of the lacking reactivity of compounds **6** towards base-induced dehydrocyanation to the 2,4-disubstituted pyrroles **7**, the reaction was attempted on substrate **6a** (R^1^ = R^2^ = Ph) under acidic conditions. Several strong and weak acids were screened at temperatures ranging from 0–150 °C. The formation of pyrrole **7a** was observed in acetic acid under microwave heating but many byproducts were formed under these conditions, presumably due to oligo- and polymerization of the product. Gratifyingly, yield and purity of pyrrole **7a** increased when **6a** was heated with microwaves to 250 °C under air cooling of the reaction vessel without any additive under solvent-free conditions. The results of the application of these conditions to cyanopyrrolines **6a**–**j** are summarized in Table 1.

**Table 1:** Microwave-assisted synthesis of 2,4-disubstituted pyrroles **7a**–**i**.

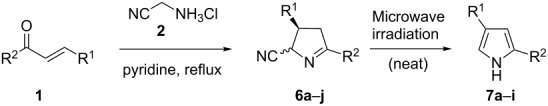					

Entry	R^1^	R^2^	Yield of **6** (%)	Yield of **7** (%)	Product

1	Ph	Ph	64^a^	81	**7a**
2	Ph	2-Naph	90^a^	83	**7b**
3	Ph	Me	49^a^	43	**7c**
4	3-NO_2_-C_6_H_4_	4-Cl-C_6_H_4_	90^a^	n.r.	**7d**
5	2,3-Cl_2_C_6_H_3_	Ph	82	66	**7e**
6	2-Br-C_6_H_4_	2-Naph	77^a^	28	**7f**
7	4-CN-C_6_H_4_	Ph	66^b^	32	**7g**
8	4-MeO-C_6_H_4_	4-F-C_6_H_4_	53^a^	61	**7h**
9	2-Cl-C_6_H_4_	4-F-C_6_H_4_	77^a^	38	**7i**
10	3,5-(CH_3_)_2_C_6_H_3_	Ph	54	not tested	**–**

^a^See [36].^b^When repeated under identical conditions, a higher yield was obtained than reported in [36] (53%).

This compound class comprises important starting materials for the preparation of the BODIPY dyes (4,4-difluoro-4-bora-3a,4a-diaza-*s*-indacenes) [37–38] as well as their aza-analogues [39], the 4,4-difluoro-4-bora-3a,4a,8-triaza-*s*-indacenes. Various methods to prepare 2,4-disubstituted pyrroles have been described in the literature. They can be obtained from enones by Michael addition of nitromethane, partial reduction and dehydrogenation of the resulting pyrroline with selenium or sulfur in moderate yields (3 steps) [40–41]. Alternatively, a Nef reaction of the nitromethane adducts gives masked 1,4-dicarbonyls which can be cyclized to compounds **7** in yields of up to 50% (over 4 steps) [42]. Other methods involve the use of stoichiometric zirconocene dichloride [43], the reductive coupling of alkynes to enones followed by ozonolysis and Paal–Knorr cyclization [21] or the reaction of vinylazides with aldehydes or ketones [23]. The most practical approach to a 2,4-disubstituted pyrrole reported to date is the recently disclosed microwave-assisted Stetter reaction of chalcone with carbohydrates as “green” formaldehyde equivalents followed by a microwave-assisted Paal–Knorr cyclization of the resulting 1,4-dicarbonyl [44]. Compared to this elegant strategy, our two-step protocol provides a higher overall yield of compound **7a** and uses less expensive reagents.

In addition to the thermal elimination of HCN, intermediates **6** can also be aromatized by dehydrogenation. In this case, the nitrile group remains in the products and 3,5-disubstituted pyrrole-2-carbonitriles **10** are obtained. Tsuge et al. reported the oxidation of pyrroline-3-carboxylates with chloranil [45] while pyrrole-2-carboxylates were obtained from the oxidation of pyrroline-2-carboxylate with chloranil [46–47] or DDQ [48]. Different oxidants were screened for the oxidation of cyanopyrroline **6a** to pyrrole **10a** and the results were judged by TLC. While chloranil, MnO_2_, NBS/DBU, and iodine/DBU in toluene or chlorobenzene did not give any conversion, bromotrichloromethane/DBU and DDQ gave positive results. The latter was found to be more suitable since the use of bromotrichloromethane/DBU resulted in many side reactions. Table 2 summarizes the results of the oxidation of **6a**–**j** with DDQ in toluene under reflux.

**Table 2:** Pyrrole-2-carbonitriles **10a**–**j**.

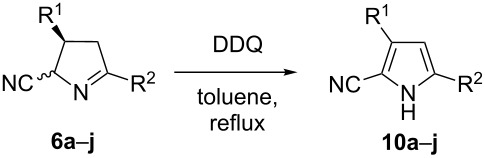				

Entry	R^1^	R^2^	Product	Yield (%)

1	Ph	Ph	**10a**	94
2	Ph	2-Naph	**10b**	59
3	Ph	Me	**10c**	–
4	3-NO_2_-C_6_H_4_	4-Cl-C_6_H_4_	**10d**	65
5	2,3-Cl_2_-C_6_H_3_	Ph	**10e**	71
6	2-Br-C_6_H_4_	2-Naph	**10f**	47
7	4-CN-C_6_H_4_	Ph	**10g**	70
8	4-MeO-C_6_H_4_	4-F-C_6_H_4_	**10h**	77
9	2-Cl-C_6_H_4_	4-F-C_6_H_4_	**10i**	71
10	3,5-(CH_3_)_2_-C_6_H_3_	Ph	**10j**	54

The reaction worked smoothly with aryl-substituted compounds. The only failure was observed in case of compound **6c** which possesses an alkyl substituent (Table 2, entry 3). As autoxidation and oxidative dealkylation of 2-alkylpyrroles can even be effected by exposure to air [49–50], the lability of **10c** towards DDQ oxidation is not very surprising.

## Conclusion

Two simple two-step procedures have been developed to convert enones to 2,4-disubstiuted pyrroles and 3,5-disubstituted pyrrole-2-carbonitriles by means of a cyclocondensation with aminoacetonitrile and a microwave-assisted dehydrocyanation or a dehydrogenation with DDQ. These methods provide a simple an efficient entry into these useful compound classes.

## Experimental

**Typical procedure for the synthesis of cyanopyrrolines 6a–j:** The cyanopyrrolines **6a**–**j** were prepared according to an earlier publication [36]. As an illustrative example, compound **6e** is prepared as follows: To a solution of (*E*)-3-(2,3-dichlorophenyl)-1-phenylprop-2-en-1-one (2.20 g, 7.94 mmol, **1e**) in pyridine (40 mL) was added aminoacetonitrile hydrochloride (1.10 g, 11.76 mmol, 1.48 equiv, **2**). The suspension was heated to reflux for 15 h. The mixture was cooled down, diluted with ethyl acetate, washed with saturated aqueous NaHCO_3_, and dried over anhydrous Na_2_SO_4_. All volatiles were removed in vacuo and the crude product was purified by column chromatography (ethyl acetate/cyclohexane 1:7) to obtain *cis*/*trans*-**6e** (2.05 g, 6.50 mmol, 82%) as a yellow oil.

**General procedure for the synthesis of 2,4-disubstituted pyrroles 7a–i:** A solution of cyanopyrroline **6a**–**i** in dichloromethane was transferred into a microwave reaction vessel. After removing the solvent in vacuo, the vessel was flushed with argon and closed with a cap. It was irradiated for 30 min (P_max_ 180 W, pressure limit 7 bar, temperatures indicated in Supporting Information File 1) in a monomode microwave apparatus under air cooling. The pressurized vessel was opened very carefully inside a well-ventilated hood (caution, hydrogen cyanide!) and the residue was purified by column chromatography.

**General procedure for the synthesis of pyrrole-2-carbonitriles 10a–j**: A round bottomed flask equipped with a magnetic stir bar was charged with cyanopyrroline **6a**–**j** and DDQ (1.15–1.20 equiv) in toluene (15–20 mL/mmol **6**). The reaction mixture was stirred under reflux until the starting material was consumed (TLC, 2–4 h). It was diluted with ethyl acetate and washed with 10% aqueous NaOH. The extracts were dried over MgSO_4_ and concentrated in vacuo to obtain the crude product which was purified by column chromatography.

## Supporting Information

File 1 Detailed experimental procedures and characterization of compounds **6e**, **6j**, **7a**–**i** and **10a**–**j** including ^1^H and ^13^C NMR spectra.
