# Supplementary material for: Simple two-step synthesis of 2,4-disubstituted pyrroles and 3,5-disubstituted pyrrole-2-carbonitriles from enones
Source: Beilstein J Org Chem. 2014 Feb 24;10:466–70. doi: 10.3762/bjoc.10.44 (PMC3943414; doi:10.3762/bjoc.10.44)
Supplement: File 1 — Detailed experimental procedures and characterization of compounds 6e, 6j, 7a–i and 10a–j including 1H and 13C NMR spectra. [file Beilstein_J_Org_Chem-10-466-s001.pdf]

Supporting Information

for

Simple two-step synthesis of 2,4-disubstituted pyrroles and 3,5-disubstituted pyrrole-2-carbonitriles from enones

Murat Kucukdisli[1], Dorota Ferenc[1], Marcel Heinz[2], Christine Wiebe[2] and Till Opatz*[1]

Address: [1]Institute of Organic Chemistry, Johannes Gutenberg University of Mainz, Duesbergweg 10–14, 55128 Mainz, Germany and [2]Department of Chemistry, University of Hamburg, Martin-Luther-King-Platz 6, 20146 Hamburg, Germany

Email: Till Opatz - opatz@uni-mainz.de

*Corresponding author

Detailed experimental procedures and characterization of compounds **6e**, **6j**, **7a**–**i** and **10a**–**j** including ^{1}H and ^{13}C NMR spectra

Content

General experimental methods.. S2
General Procedures S3
Analytical data S4
References S15
^{1}H and ^{13}C NMR spectra.. S16

General experimental methods

All reactions were carried out in dried glassware under an inert atmosphere (argon) in anhydrous solvents using standard syringe and septa techniques. The solvents used for chromatography were distilled prior to use. All other solvents and reagents were purchased from commercial suppliers and were used without further purification. TLC experiments were carried out on aluminum sheets coated with silica gel 60 F_{254} and spots were visualized with UV-light (254 nm) and developed with Seebach's reagent or *p*-anisaldehyde and heating. Column chromatography was carried out on silica gel (32–63 μm, 60 Å, 230–400 mesh). Melting points were determined with a Dr. Tottoli apparatus and are uncorrected. NMR spectra were recorded with a 300, 400 and 500 MHz spectrometer. The spectra were measured in $CDCl_3$ or DMSO-d_6 at ambient temperature unless otherwise stated and the chemical shifts were referenced to the residual solvent signal ($CDCl_3$: δ H = 7.26 ppm, δ C = 77.16 ppm; DMSO-d_6: δ H = 2.50 ppm, δ C = 39.52 ppm). IR spectra were recorded on routine FTIR spectrometers using a diamond ATR unit or a NaCl pellet. For high resolution FAB-MS (FAB-HRMS), PEG 300 or PEG 600 was used as internal standard. ESI-HRMS spectra were recorded on a Q-TOF instrument with a dual source and a suitable external calibrant. Microwave reactions were carried out in a CEM Discover instrument.

The cyanopyrrolines **6a–j** were prepared according to the literature from commercially available aminoacetonitrile hydrochloride and chalcones [1].

General procedures

General procedure A: synthesis of 2,4-disubstituted pyrroles 7a–i: A solution of cyanopyrrolines **6a**–**i** in dichloromethane was transferred into a microwave reaction vessel. After removing the solvent in vacuo, the vessel was flushed with argon and closed with a cap. It was irradiated for 30 min (P_{max} 180 W) in a monomode microwave apparatus under air cooling. The temperatures reached (IR sensor) are listed in Table S1. The pressurized vessel was opened very carefully inside a well-ventilated hood (caution, hydrogen cyanide!) and the residue was purified by column chromatography.

Table S1: Temperature parameters of the microwave experiments

Entry	Product	T_{set} (°C)	T_{max}
1	**7a**	250	190
2	**7b**	250	230
3	**7c**	250	150
4	**7d**	250	195
5	**7e**	250	220
6	**7f**	250	200
7	**7g**	250	230
8	**7h**	250	180
9	**7i**	250	160

General procedure B: synthesis of pyrrole-2-carbonitriles 10a–j: A round bottomed flask equipped with a magnetic stir bar was charged with cyanopyrroline **6a**–**j** and DDQ (1.15–1.20 equiv) in toluene (15–20 mL/mmol **6**). The reaction mixture was stirred under reflux until the starting material was consumed (TLC, 2–4 h). It was diluted with ethyl acetate and washed with 10% aqueous NaOH. The extracts were

dried over $MgSO_4$ and concentrated in vacuo to obtain the crude product which was purified by column chromatography.

Analytical data

3-(2,3-Dichlorophenyl)-5-phenyl-3,4-dihydro-2*H*-pyrrole-2-carbonitrile (**6e**): The title compound was prepared according to literature [1]. R_f 0.16 (ethyl acetate/cyclohexane 1:7); IR (NaCl) $\tilde{\nu}$ 3062, 2924, 2243, 1677, 1610, 1574, 1449, 1421, 1345, 1263, 1182, 1152, 1045, 1025, 922, 763, 691 cm^{-1}; ^1H NMR (400 MHz, $CDCl_3$) δ 8.20–6.90 (m, 14H, H_{Ar} , *trans and cis*), 5.50 (dt, *J* = 8.4, 1.2 Hz, 0.5H, H-2, *cis*), 5.10 (dt, *J* = 5.6, 1.6 Hz, 1H, H-2, *trans*), 4.53 (dt, *J* = 8.5, 7.5 Hz, 0.5H, H-3, *cis*), 4.44 (dt, *J* = 9.7, 5.7 Hz, 1H, H-3, *trans*), 3.72 (ddd, *J* = 17.6, 9.7, 1.8 Hz, 1H, H-4a, *trans*), 3.50 (ddd, *J* = 17.2, 8.7, 1.2 Hz, 0.5H, H-4a, *cis*), 3.43 (ddd, *J* = 17.2, 7.5, 1.5 Hz, 0.5H, H-4b, *cis*), 3.28 (ddd, *J* = 17.6, 5.8, 1.4 Hz, 1H, H-4b, *trans*) ppm; APT NMR (101 MHz, $CDCl_3$) δ 140.3 (C_q), 132.3 (CH_{Ar}, *cis*), 132.3 (CH_{Ar}, *trans*), 130.3 (CH_{Ar}, *cis*), 130.1 (CH_{Ar}, *trans*), 129.0 (C3'',5'', cis), 128.97 (C3'',5'', *trans*), 128.5 (C2'',6'', *trans*), 128.4 (C2'',6'', *cis*), 128.1 (CH_{Ar}, *trans*), 128.0 (CH_{Ar}, *cis*), 126.0 (CH_{Ar}, *cis*), 125.8 (CH_{Ar}, *trans*), 118.7 (CN), 67.3 (C-2, *trans*), 65.7 (C2, cis), 46.2 (C3, *trans*), 43.6 (C3, *cis*), 43.0 (C4, *trans*), 41.2 (C4, *cis*) ppm; HRMS (FAB) calcd for $[C_{17}H_{12}Cl_2N_2 + H]^+$ 315.0460, found 315.0456.

3-(3,5-Dimethylphenyl)-5-phenyl-3,4-dihydro-2*H*-pyrrole-2-carbonitrile (**6j**): The title compound was prepared according to literature [1]. To a solution of (*E*)-3-(3,5-dimethylphenyl)-1-phenylprop-2-en-1-one (471 mg, 2.00 mmol, **1j**) in pyridine (6 mL) was added aminoacetonitrile hydrochloride (284 mg, 3.07 mmol, 1.54 equiv, **2**). The suspension was heated to reflux. The reaction was monitored by TLC and several portions of compound **2** were added: 148 mg, 1.60 mmol after 2.5 h; 92 mg,

1.00 mmol after 4 h; 103 mg, 1.11 mmol after 20 h; 60 mg, 0.65 mmol after 22 h; 63 mg, 0.68 mmol after 24 h; 51 mg, 0.55 mmol after 25 h. After a total time of 26 h stirring at reflux, the mixture was cooled, diluted with ethyl acetate, washed with saturated aqueous $NaHCO_3$, and dried over anhydrous Na_2SO_4. All volatiles were removed in vacuo and the crude product was purified by column chromatography (ethyl acetate/cyclohexane 1:8) to obtain *trans*-**6j** (188 mg, 0.69 mmol, 34%) as a brown oil and *cis*-**6j** (111 mg, 0.41 mmol, 20%). Analytical data of the *trans*-isomer: R_f 0.26 (ethyl acetate/cyclohexane 1:8); IR (NaCl) $\tilde{\nu}$ 3059, 3018, 2918, 2244, 1682, 1607, 1575, 1495, 1448, 1468, 1345, 1264, 1179, 1050, 1026, 948, 871, 846, 763, 691 cm^{-1}; ^1H NMR (400 MHz, $CDCl_3$) δ 7.97–7.87 (m, 2H, H-2'',6''), 7.58–7.42 (m, 3H, H-3'',5'', H-4''), 6.96–6.90 (m, 1H, H-4'), 6.85 (s, 2H, H-2',6'), 4.91 (dt, J = 7.1, 1.9 Hz, 1H, H-2), 3.85 (d-pseudo-t, J ≈ 9.5, 7.3 Hz, 1H, H-3), 3.65 (ddd, J = 17.5, 9.5, 1.9 Hz, 1H, H-4a), 3.24 (ddd, J = 17.5, 7.5, 1.9 Hz, 1H, H-4b), 2.29 (s, 6H, CH_3) ppm; APT NMR (126 MHz, $CDCl_3$) δ = 177.0 (C5), 140.2 (C_q), 139.1 (C3'',5''), 132.8 (C_q), 132.2 (CH_{Ar}), 129.6 (CH_{Ar}), 128.9 (CH_{Ar}, 2C), 128.5 (CH_{Ar}, 2C), 124.6 (CH_{Ar}, 2C), 119.4 (CN), 69.1 (C2), 48.8 (C3), 44.0 (C4), 21.4 (2 CH_3) ppm; HRMS (FAB) calcd for $[C_{19}H_{18}N_2 + H]^+$ 275.1548, found 275.1548.

2,4-Diphenyl-1*H*-pyrrole (**7a**): The title compound was prepared from **6a** (123 mg, 0.50 mmol) according to general procedure A described above. The product was purified by column chromatography (ethyl acetate/cyclohexane 1:4) to obtain **7a** (89 mg, 0.41 mmol, 81%) as a white solid: mp 178–179 °C (lit. [2] 178–180 °C); R_f 0.38 (ethyl acetate/cyclohexane 1:4); IR (ATR) $\tilde{\nu}$ 3440, 3029, 1605, 1491, 1453, 1134, 808, 752, 691 cm^{-1}; ^1H NMR, COSY (400 MHz, DMSO-d_6) δ 11.44 (br s, 1H, NH), 7.70–7.66 (m, 2H, H-2',6'), 7.63–7.58 (m, 2H, H-2'',6''), 7.40–7.35 (m, 2H, H-3',5'), 7.35–7.30 (m, 3H, H-5, H-3'',5''), 7.20–7.15 (m, 1H, H-4'), 7.15–7.10 (m, 1H, H-

4''), 6.96 (dd, J = 2.5, 1.9 Hz, 1H, H-3) ppm; ^{13}C NMR, HSQC, HMBC (101 MHz, DMSO-d_6) δ 135.7 (C1''), 132.7 (C1'), 132.2 (C2), 128.7 (C3',5'), 128.5 (C3',5'), 125.7 (C4'), 125.0 (C4''), 124.7 (C4), 124.4 (C2'',6''), 123.4 (C2',6'), 116.6 (C5), 103.2 (C3) ppm; HRMS (ESI) calcd for $[C_{16}H_{13}N + H]^+$ 220.1126, found 220.1126.

2-(Naphthalen-2-yl)-4-phenyl-1*H*-pyrrole (**7b**): The title compound was prepared from **6b** (207 mg, 0.70 mmol) according to general procedure A described above. The product was purified by column chromatography (ethyl acetate/cyclohexane 1:10) to obtain **7b** (157 mg, 0.58 mmol, 83%) as a pale yellow solid: mp 219–220 °C (lit. [3] 222–224 °C); R_f 0.16 (ethyl acetate/cyclohexane 1:10); IR (ATR) $\tilde{\nu}$ 3390, 3027, 1602, 1451, 1263, 1128, 856, 824, 802, 743, 690 cm^{-1}; ^{1}H NMR, COSY (400 MHz, DMSO-d_6) δ 11.64 (br s, 1H, NH), 8.17 (s, 1H, H-1'), 7.93–7.89 (m, 2H, H-3', H-4'), 7.89–7.83 (m, 2H, H-5', H-8'), 7.67–7.63 (m, 2H, H-2'',6''), 7.52–7.48 (m, 1H, H_{Naphth}), 7.46–7.41 (m, 2H, H_{Naphth}, H-5), 7.34 (pseudo-t, J_{app} ≈ 7.7 Hz, 2H, H-3'',5''), 7.17–7.10 (m, 2H, H-4'', H-3) ppm; ^{13}C NMR, HSQC, HMBC (101 MHz, DMSO-d_6) δ 135.7 (C1''), 133.4 (C_{Naphth}), 132.2 (C2), 131.5 (C_{Naphth}), 130.2 (C2'), 128.6 (C3'',5''), 128.2 (C4'), 127.6 (CH_{Naphth}), 127.5 (CH_{Naphth}), 126.5 (CH_{Naphth}), 125.2 (CH), 125.1 (CH), 124.9 (C4), 124.4 (C2'',6''), 123.1 (C3'), 120.5 (C1'), 117.2 (C5), 104.1 (C3) ppm; HRMS (ESI) calcd for $[C_{20}H_{15}N + H]^+$ 270.1283, found 270.1273.

2-Methyl-4-phenyl-1*H*-pyrrole (**7c**): The title compound was prepared from **6c** (92 mg, 0.50 mmol) according to general procedure A described above. The product was purified by column chromatography (ethyl acetate/cyclohexane 1:4) to obtain **7c** (34 mg, 0.21 mmol, 43%) as a yellow oil: R_f 0.26 (ethyl acetate/cyclohexane 1:4); IR (ATR) $\tilde{\nu}$ 3412, 3370, 3027, 2923, 1603, 1529, 1449, 1123, 795, 762, 697 cm^{-1}; ^{1}H NMR (400 MHz, DMSO-d_6) δ 10.64 (br s, 1H, NH), 7.49–7.44 (m, 2H, H-2',6'), 7.28–7.23 (m, 2H, H-3',5'), 7.08–7.03 (m, 1H, H-4'), 7.02 (dd, J = 2.8, 1.8 Hz, 1H, H-5),

6.12–6.10 (m, 1H, H-3), 2.19 (d, ^{4}J = 1.0 Hz, 3H, CH_3) ppm; ^{13}C NMR, HSQC, HMBC (101 MHz, DMSO-d_6) δ 136.5 (C1'), 128.5 (C3',5'), 128.1 (C2), 124.5 (C4'), 124.2 (C2',6'), 123.2 (C4), 113.3 (C5), 103.3 (C3), 12.8 (CH_3) ppm; HRMS (ESI) calcd for $[C_{11}H_{12}N + H]^+$ 158.0970, found 158.0956.

4-(2,3-Dichlorophenyl)-2-phenyl-1*H*-pyrrole (**7e**): The title compound was prepared from **6e** (172 mg, 0.55 mmol) according to general procedure A described above. The product was purified by column chromatography (ethyl acetate/cyclohexane 1:4) to obtain **7e** (104 mg, 0.36 mmol, 66%) as a white solid: mp 122–123 °C; R_f 0.40 (ethyl acetate/cyclohexane 1:4); IR (ATR) $\tilde{\nu}$ 3431, 3096, 3058, 1605, 1584, 1482, 1455, 1413, 1121, 1036, 814, 774, 756, 729, 692 cm^{-1}; ^{1}H NMR, COSY (400 MHz, DMSO-d_6) δ 11.65 (br s, 1H, NH), 7.71–7.66 (m, 2H, H-2',6'), 7.58 (dd, *J* = 7.8, 1.6 Hz, 1H, H-6''), 7.47 (dd, *J* = 8.0, 1.6 Hz, 1H, H-4''), 7.41–7.31 (m, 4H, H-3',5', H-5, H-5''), 7.20 (pseudo-tt, J_{app} ≈ 7.4, 1.5 Hz, 1H, H-4'), 6.93 (dd, *J* = 2.6, 1.8 Hz, 1H, H-3) ppm; ^{13}C NMR, HSQC, HMBC (101 MHz, DMSO-d_6) δ 136.9 (C1''), 132.6 (C3''), 132.3 (C1'), 131.5 (C2), 128.8 (C3',5'), 128.7 (C6''), 128.4 (C2''), 128.0 (C5''), 127.4 (C4''), 126.0 (C4'), 123.6 (C2',6'), 121.5 (C4), 120.0 (C5), 106.3 (C3) ppm; HRMS (ESI) calcd for $[C_{16}H_{11}NCl_2 + H]^+$ 288.0347, found 288.0354.

4-(2-Bromophenyl)-2-(naphthalen-2-yl)-1*H*-pyrrole (**7f**): The title compound was prepared from **6f** (329 mg, 0.88 mmol) according to general procedure A described above. The product was purified by column chromatography (ethyl acetate/cyclohexane 1:5) to obtain **7f** (87 mg, 0.25 mmol, 28%) as a white solid: mp 121–122 °C; R_f 0.33 (ethyl acetate/cyclohexane 1:5); IR (ATR) $\tilde{\nu}$ 3432, 3054, 1603, 1508, 1466, 1418, 1267, 1122, 855, 810, 749 cm^{-1}; ^{1}H NMR, COSY (400 MHz, DMSO-d_6) δ 11.75 (br s, 1H, NH), 8.17 (s, 1H, H-1'), 7.94–7.84 (m, 4H, 4 H_{Naphth}), 7.67 (dd, *J* = 8.0, 1.2 Hz, 1H, H-3''), 7.59 (dd, *J* = 7.8, 1.7 Hz, 1H, H-6''), 7.54–7.47

(m, 1H, H_{Naphth}), 7.47–7.36 (m, 2H, H_{Naphth}, H-5''), 7.34 (dd, *J* = 2.9, 1.7 Hz, 1H, H-5), 7.15 (ddd, *J* = 8.9, 8.0, 1.7 Hz, 1H, H-4''), 7.04 (dd, *J* = 2.8, 1.7 Hz, 1H, H-3) ppm; ^{13}C NMR, HSQC, HMBC (101 MHz, DMSO-d_6) δ 136.4 (C1''), 133.47 (C3''), 133.45, 131.5, 131.2 (C2), 130.7 (C6''), 130.0, 128.3, 127.8, 127.7, 127.5, 127.4, 126.5, 125.3, 123.5 (C4), 123.1, 121.1 (C2''), 120.6 (C1'), 119.8 (C5), 107.2 (C3) ppm; HRMS (ESI) calcd for $[C_{20}H_{14}NBr + H]^+$ 348.0388, found 348.0398.

4-(5-Phenyl-1*H*-pyrrol-3-yl)benzonitrile (**7g**): The title compound was prepared from **6g** (282 mg, 1.04 mmol) according to general procedure A described above. The product was purified by column chromatography (ethyl acetate/cyclohexane 1:6) to obtain **7g** (81 mg, 0.33 mmol, 32%) as a pale yellow solid: mp 205–207 °C; R_f 0.29 (ethyl acetate/cyclohexane 1:6); IR (ATR) $\tilde{\nu}$ 3353, 3052, 2223, 1602, 1492, 1149, 926, 848, 809, 776, 753, 695 cm^{-1}; ^{1}H NMR, COSY (400 MHz, DMSO-d_6) δ 11.70 (br s, 1H, NH), 7.86–7.79 (AA' part of AA'BB' system, 2H, H-3,5), 7.77–7.73 (BB' part of AA'BB' system, 2H, H-2,6), 7.71–7.68 (m, 1H, H-2'',6''), 7.59 (dd, *J* = 2.8, 1.8 Hz, 1H, H-2'), 7.39 (pseudo-t, J_{app} ≈ 7.8 Hz, 2H, H-3',5'), 7.23–7.18 (m, 1H, H-4''), 7.09 (dd, *J* = 2.4, 1.8 Hz, 1H, H-4') ppm; ^{13}C NMR, HSQC, HMBC (101 MHz, DMSO-d_6) δ 140.6 (C4), 133.0 (C5'), 132.6 (C2,6), 132.3 (C1''), 128.8 (C3'',5''), 126.1 (C4''), 124.8 (C3,5), 123.6 (C2'',6''), 123.1 (C3'), 119.5 (CN), 118.9 (C2'), 106.7 (C1), 103.5 (C4') ppm; HRMS (ESI) calcd for $[C_{17}H_{12}N_2 + H]^+$ 245.1079, found 245.1078.

2-(4-Fluorophenyl)-4-(4-methoxyphenyl)-1*H*-pyrrole (**7h**): The title compound was prepared from **6h** (223 mg, 0.76 mmol) according to general procedure A described above. The product was purified by column chromatography (ethyl acetate/cyclohexane 1:8) to obtain **7h** (123 mg, 0.46 mmol, 61%) as a white solid: mp 138–139 °C; R_f 0.11 (ethyl acetate/cyclohexane 1:8); IR (ATR) $\tilde{\nu}$ 3443, 3427, 3008, 2960, 2840, 1572, 1505, 1439, 1246, 1036, 835, 798 cm^{-1}; ^{1}H NMR, COSY (400

MHz, DMSO-d_6) δ 11.32 (br s, 1H, NH), 7.71–7.66 (m, 2H, H-2',6'), 7.53–7.49 (AA' part of AA'BB' system, 2H, H-2'',6''), 7.24–7.17(m, 3H, H-3',5', H-5), 6.91–6.88 (BB' part of AA'BB' system, 2H, H-3'',5''), 6.84 (dd, *J* = 2.7, 1.7 Hz, 1H, H-3), 3.75 (s, 3H, CH_3) ppm; ^{13}C NMR, HSQC, HMBC (101 MHz, DMSO-d_6) δ 160.4 (d, $^1J_{C,F}$ = 242.4 Hz, C4'), 157.1 (C4''), 131.1 (C2), 129.5 (d, $^4J_{C,F}$ = 2.9 Hz, C1'), 128.4 (C1''), 125.5 (C3'',5''), 125.2 (d, $^3J_{C,F}$ = 7.8 Hz, C2',6'), 124.6 (C4), 115.6 (C5, overlapped with the doublet of C3',5'), 115.5(d, $^2J_{C,F}$ = 21.8 Hz, C3',5'), 114.0 (C3'',5''), 103.0 (C3), 55.0 (CH_3) ppm; HRMS (ESI) calcd for $[C_{17}H_{14}NOF + H]^+$ 268.1138, found 268.1129.

4-(2-Chlorophenyl)-2-(4-fluorophenyl)-1*H*-pyrrole (**7i**): The title compound was prepared from **6i** (224 mg, 0.75 mmol) according to general procedure A described above. The product was purified by column chromatography (ethyl acetate/cyclohexane 1:6) to obtain **7i** (78 mg, 0.29 mmol, 38%) as a white solid: mp 124–126 °C; R_f 0.24 (ethyl acetate/cyclohexane 1:6); IR (ATR) $\tilde{\nu}$ 3434, 3267, 3065, 1661, 1492, 1230, 1097, 930, 835, 810, 753 cm^{-1}; ^{1}H NMR, COSY (400 MHz, DMSO-d_6) δ 11.58 (br s, 1H, NH), 7.74–7.68 (m, 2H, H-2',6'), 7.61 (dd, *J* = 7.8, 1.7 Hz, 1H, H-6''), 7.46 (dd, *J* = 8.0, 1.3 Hz, 1H, H-3''), 7.36–7.30 (m, 2H, H-5'', H-5), 7.26–7.17 (m, 3H, H-3',5', H-4''), 6.90 (dd, *J* = 2.6, 1.8 Hz, 1H, H-3) ppm; ^{13}C NMR, HSQC, HMBC (101 MHz, DMSO-d_6) δ 160.6 (d, $^1J_{C,F}$ = 242.5 Hz, C4'), 134.2 (C1''), 130.5 (C2), 130.3 (C2''), 130.2 (C3''), 130.0 (C6''), 129.2 (d, $^4J_{C,F}$ = 3.1 Hz, C1'), 127.3 (C5''), 126.8 (C4''), 125.4 (d, $^3J_{C,F}$ = 7.9 Hz, C2',6'), 121.7 (C4), 119.5 (C5), 115.6 (d, $^2J_{C,F}$ = 21.5 Hz, C3',5'), 106.0 (C3) ppm; HRMS (ESI) calcd for $[C_{16}H_{11}NFCl + H]^+$ 272.0642, found 272.0634.

3,5-Diphenyl-1*H*-pyrrole-2-carbonitrile (**10a**): The title compound was prepared from **6a** (292 mg, 1.19 mmol) and DDQ (310 mg, 1.36 mmol) in 8 mL of toluene according to general procedure B described above. The crude product was purified

by column chromatography (ethyl acetate/cyclohexane 1:10) to obtain **10a** (273 mg, 1.12 mmol, 94%) as a white solid: mp 192–193 °C (lit. [4] 194–195 °C); R_f 0.23 (ethyl acetate/cyclohexane 1:10); ^{1}H NMR (400 MHz, DMSO-d$_6$) δ 12.75 (br s, 1H, NH), 7.83–7.78 (m, 2H, H-2',6' or H-2'',6''), 7.76–7.72 (m, 2H, H-2',6'or H-2'',6''), 7.51–7.43 (m, 4H, H-3',5', H-3'',5''), 7.39–7.32 (m, 2H, H-4', H-4''), 7.13 (s, 1H, H-4) ppm; ^{13}C-NMR, DEPT, HSQC, HMQC (101 MHz, DMSO-d$_6$) δ 137.2 (C5), 134.8 (C3), 132.6, 130.4 (C1', C1''), 129.0, 128.9 (4C, C3',5', C3'',5''), 127.9, 127.6 (C4', C4''), 126.2, 124.8 (C2',6', C2'',6''), 115.5 (CN), 106.1 (C4), 97.2 (C2) ppm; HRMS (FAB) calcd for $[C_{17}H_{12}N_2 + H]^+$ 245.1000, found 245.1000; EA ($C_{17}H_{12}N_2$): calcd. 83.58% C, 4.95% H, 11.47% H; found 83.47% C, 5.27% H, 11.14% N.

5-(Naphthalen-2-yl)-3-phenyl-1*H*-pyrrole-2-carbonitrile (**10b**): The title compound was prepared from **6b** (207 mg, 0.70 mmol) and DDQ (191 mg, 0.84 mmol) in 14 mL of toluene according to general procedure B described above. The crude product was purified by column chromatography (ethyl acetate/cyclohexane 1:3) to obtain **10b** (121 mg, 0.41 mmol, 59%) as a white solid; mp 227–229 °C; R_f 0.47 (ethyl acetate/cyclohexane 1:3); IR (ATR) $\tilde{\nu}$ 3268, 3056, 2213, 1457, 1230, 863, 853, 808, 764 cm^{-1}; ^{1}H NMR, COSY (400 MHz, DMSO-d$_6$) δ 12.93 (br s, 1H, NH), 8.36 (d, J = 1.7 Hz, 1H, H-1''), 8.01 (d, J = 8.6 Hz, 1H, H-4''), 7.97 (dd, J = 8.6, 1.7 Hz, 1H, H-3''), 7.94–7.91 (m, 2H, H_{Naphth}), 7.80–7.76 (m, 2H, H-2',6'), 7.59–7.47 (m, 4H, H-3',5', 2H_{Naphth}), 7.40–7.35 (m, 1H, H-4'), 7.29 (d, J = 2.7 Hz, 1H, H-4) ppm; ^{13}C NMR (101 MHz, DMSO-d$_6$) δ 137.2, 134.9, 133.0, 132.6, 132.4, 129.0 (C3',5'), 128.6 (C4''), 127.96, 127.92, 127.73, 127.72, 126.9, 126.4, 126.3 (C2',6'), 123.23 (C1''), 123.20 (C3''), 115.6 (CN), 106.8 (C4), 97.5 (C2) ppm; HRMS (ESI) calcd. for $[C_{21}H_{14}N_2 + H]^+$ 295.1235, found 295.1228.

5-(4-Chlorophenyl)-3-(3-nitrophenyl)-1*H*-pyrrole-2-carbonitrile (**10d**)**:** The title compound was prepared from **6d** (302 mg, 0.93 mmol) and DDQ (252 mg, 1.11 mmol) in 20 mL of toluene according to general procedure B described above. The crude product was purified by column chromatography (ethyl acetate/cyclohexane 1:6) to obtain **10d** (195 mg, 0.60 mmol, 65%) as a white foam: R_f 0.14 (ethyl acetate/cyclohexane 1:6); IR (ATR) $\tilde{\nu}$ 3299, 2925, 2212, 1533, 1350, 1263, 1094, 799, 740 cm^{-1}; ^{1}H NMR (400 MHz, DMSO-d_6) δ 13.06 (br s, 1H, NH), 8.56 (t, J = 2.0 Hz, 1H, H-2'), 8.23–8.17 (m, 2H, H-4', H-6'), 7.88–7.83 (AA' part of AA'BB' system, 2H, H-2'',6''), 7.80 (t, J = 8.0 Hz, 1H, H-5'), 7.59–7.53 (BB' part of AA'BB' system, 2H, H-3'',5''), 7.41 (s, 1H, H-4) ppm; ^{13}C NMR, HSQC, HMBC (101 MHz, DMSO-d_6) δ 148.4 (C3'), 136.4 (C5), 134.1 (C1'), 132.7 (C4''), 132.4 (C6'), 132.2 (C3), 130.7 (C5'), 129.13 (C1''), 129.09 (C3'',5''), 126.6 (C2'',6''), 122.3 (C4'), 120.4 (C2'), 114.9 (CN), 107.2 (C4), 98.2 (C2) ppm; HRMS (ESI) calcd. for $[C_{17}H_{10}N_3O_2Cl + H]^+$ 346.0359, found 346.0367.

3-(2,3-Dichlorophenyl)-5-phenyl-1*H*-pyrrole-2-carbonitrile (**10e**): The title compound was prepared from **6e** (158 mg, 0.50 mmol) and DDQ (136 mg, 0.60 mmol) in 10 mL of toluene according to general procedure B described above. The crude product was purified by column chromatography (ethyl acetate/cyclohexane 1:5) to obtain **10e** (111 mg, 0.35 mmol, 71%) as a yellow solid: mp 204–205 °C; R_f 0.21 (ethyl acetate/cyclohexane 1:10); IR (ATR) $\tilde{\nu}$ 3290, 2207, 1586, 1483, 1447, 1395, 1264, 1191, 1109, 1056, 1020, 820, 785, 774, 754, 684 cm^{-1}; ^{1}H NMR (400 MHz, $CDCl_3$) δ 9.26 (br s, 1H, NH), 7.56–7.52 (m, 2H, H-2'',6''), 7.49 (dd, J = 8.0, 1.6 Hz, 1H, H-4'), 7.47–7.41 (m, 2H. H-3'',5''), 7.40 (dd, J = 7.7, 1.6 Hz, 1H, H-2'), 7.38–7.33 (m, 1H, H-4''), 7.27 (dd, J = 8.0, 7.7 Hz, 1H, H-3'), 6.75 (d, J = 2.8 Hz, 1H, H-4) ppm; ^{13}C NMR (101 MHz, DMSO-d_6) δ 136.5 (C5), 134.2 (C3),

132.5, 132.2, 130.3 (2 C_q), 130.18, 130.15, 129.0, 128.3, 128.0, 124.8, 114.1 (CN), 108.7 (C4), 100.1 (C2) ppm; HRMS (FAB) calcd for $[C_{17}H_{10}Cl_2N_2 + H]^+$ 313.0307, found: 313.0299.

3-(2-Bromophenyl)-5-(naphthalen-2-yl)-1*H*-pyrrole-2-carbonitrile (**10f**): The title compound was prepared from **6f** (504 mg, 1.34 mmol) and DDQ (366 mg, 1.61 mmol) in 25 mL of toluene according to general procedure B described above. The crude product was purified by column chromatography (ethyl acetate/cyclohexane 1:5) to obtain **10f** (234 mg, 0.63 mmol, 47%) as a white solid: mp 180–181 °C; R_f 0.26 (ethyl acetate/cyclohexane 1:5); IR (ATR) $\tilde{\nu}$ 3264, 3056, 2214, 1507, 1446, 1265, 812, 757, 725 cm^{-1}; ^1H NMR, COSY (400 MHz, DMSO-d_6) δ 13.05 (br d, *J* = 2.0 Hz, 1H, NH), 8.35 (d, *J* = 1.1 Hz, 1H, H-1’’), 8.00 (d, *J* = 8.7 Hz, 1H, H-4’’), 7.96–7.90 (m, 3H, H-3’’, 2 H_{Naphth}), 7.79 (dd, *J* = 8.0, 0.8 Hz, 1H, H-3’), 7.59–7.47 (m, 4H, 2 H_{Naphth}, H-5’, H-6’), 7.36 (ddd, *J* = 8.0, 6.9, 2.3 Hz, 1H, H-4’), 7.05 (d, *J* = 2.6 Hz, 1H, H-4) ppm; ^{13}C NMR, HSQC, HMBC (101 MHz, DMSO-d_6) δ 136.2 (C5), 134.6 (C3), 133.9 (C1’), 133.2 (C3’), 133.1 (C8a’’), 132.4 (C4a’’), 131.7 (C6’), 130.0 (C4’), 128.7 (C4’’), 128.0 (CH), 127.96 (CH), 127.9 (C2’’), 127.7 (CH), 126.9, 126.4 (C6’’, C7’’), 123.24, 123.18 (C1’’, C3’’), 122.6 (C2’), 114.5 (CN), 109.3 (C4), 100.3 (C2) ppm; HRMS (ESI) calcd for $[C_{21}H_{13}N_2Br + Na]^+$ 395.0160, found 395.0154.

3-(4-Cyanophenyl)-5-phenyl-1*H*-pyrrole-2-carbonitrile (**10g**): The title compound was prepared from **6g** (310 mg, 1.14 mmol) and DDQ (311 mg, 1.37 mmol) in 20 mL of toluene according to general procedure B described above. The crude product was purified by column chromatography (ethyl acetate/cyclohexane 1:4) to obtain **10g** (216 mg, 0.80 mmol, 70%) as a yellow solid: mp 228–229 °C; R_f 0.15 (ethyl acetate/cyclohexane 1:4); IR (ATR) $\tilde{\nu}$ 3294, 3065, 2224, 2204, 1606, 1263, 840, 813,

758, 730, 689 cm^{-1}; ^{1}H NMR (400 MHz, DMSO-d_6) δ 13.01 (br s, 1H, NH), 7.98–7.95 (AA' part of AA'BB' system, 2H, H-3',5'), 7.94–7.91 (BB' part of AA'BB' system, 2H, H-2',6'), 7.83–7.79 (m, 2H, H-2'',6''), 7.50–7.45 (pseudo-t, J_{app} ≈ 7.7 Hz, 2H, H-3'',5''), 7.38–7.33 (pseudo-tt, J_{app} ≈ 1.2, 7.4 Hz, 1H, H-4''), 7.28 (d, J = 2.7 Hz, 1H, H-4) ppm; ^{13}C NMR, HSQC, HMBC (101 MHz, DMSO-d_6) δ 137.6 (C5), 137.2 (C1'), 133.0 (C3',5'), 132.6 (C3), 130.2 (C1''), 129.1 (C3'',5''), 128.2 (C4''), 126.8 (C2',6'), 124.9 (C2'',6''), 118.8 (NC-C4'), 115.1 (NC-C2), 109.9 (C4'), 106.7 (C4), 98.1(C2) ppm; HRMS (ESI) calcd for $[C_{18}H_{11}N_3 + Na]^+$ 292.0851, found 292.0861.

5-(4-Fluorophenyl)-3-(4-methoxyphenyl)-1*H*-pyrrole-2-carbonitrile (**10h**): The title compound was prepared from **6h** (293 mg, 1.00 mmol) and DDQ (271 mg, 1.20 mmol) in 20 mL of toluene according to general procedure B described above. The crude product was purified by column chromatography (ethyl acetate/cyclohexane 1:4) to obtain **10h** (225 mg, 0.77 mmol, 77%) as a white solid: mp 238–239 °C; R_f 0.26 (ethyl acetate/cyclohexane 1:4); IR (ATR) $\tilde{\nu}$ 3298, 2927, 2839, 2206, 1599, 1507, 1350, 1254, 1226, 1166, 840, 809 cm^{-1}; ^{1}H NMR, COSY (400 MHz, DMSO-d_6) δ 12.64 (s, 1H, NH), 7.87–7.80 (m, 2H, H-2'',6''), 7.69–7.64 (AA' part of AA'BB' system, 2H, H-2',6'), 7.35–7.28 (m, 2H, H-3'',5''), 7.09–7.02 (m, 3H, H-4, H-3',5'), 3.80 (s, 3H, CH_3) ppm; ^{13}C NMR, HSQC, HMBC (101 MHz, DMSO-d_6) δ 161.7 (d, $^{1}J_{CF}$= 245.3 Hz, C4''), 158.9 (C4'), 136.2 (C5), 134.8 (C3), 127.5 (C2',6'), 127.2 (d, $^{4}J_{CF}$= 3.2 Hz, C1''), 127.0 (d, $^{3}J_{CF}$= 8.3 Hz, C2'',6''), 125.0 (C1''), 116.0 (d, $^{2}J_{CF}$= 21.7 Hz, C3'',5''), 115.8 (CN), 114.4 (C3',5'), 105.8 (C4), 96.5 (C2), 55.2 (CH_3) ppm; HRMS (ESI) calcd for $[C_{18}H_{13}N_2OF + Na]^+$ 315.0910, found 315.0912.

3-(2-Chlorophenyl)-5-(4-fluorophenyl)-1*H*-pyrrole-2-carbonitrile (**10i**): The title compound was prepared from **6i** (149 mg, 0.50 mmol) and DDQ (136 mg,

0.60 mmol) in 10 mL of toluene according to general procedure B described above. The crude product was purified by column chromatography (ethyl acetate/cyclohexane 1:6) to obtain **10i** (105 mg, 0.354 mmol, 71%) as a white solid: mp 214–216 °C; R_f 0.20 (ethyl acetate/cyclohexane 1:6); IR (ATR) $\tilde{\nu}$ 3290, 3068, 2926, 2214, 1600, 1508, 1235, 1158, 841, 805, 761, 749 cm^{-1}; ^1H NMR, COSY (300 MHz, DMSO-d_6) δ 12.88 (br s, 1H, NH), 7.88–7.79 (m, 2H, H-2'',6''), 7.64–7.58 (m, 1H, $H_{Ar'}$), 7.54–7.49 (m, 1H, $H_{Ar'}$), 7.47–7.42 (m, 2H, $H_{Ar'}$), 7.36–7.26 (m, 2H, H-3'',5''), 6.91 (s, 1H, H-4) ppm; ^{13}C NMR, HSQC, HMBC (75 MHz, DMSO-d_6) δ 161.8 (d, $^1J_{CF}$= 245.7 Hz, C4''), 135.5 (C5), 132.7 (C3), 131.7 (C_q), 131.6 (C_q), 130.0 ($CH_{Ar'}$), 129.8 ($CH_{Ar'}$), 127.5 ($CH_{Ar'}$), 127.15 (C1''), 127.06 (d, $^3J_{CF}$= 8.1 Hz, C2'',6''), 116.0 (d, $^2J_{CF}$= 21.7 Hz, C3'',5''), 114.5 (CN), 108.8 (C4), 100.0 (C2) ppm; HRMS (ESI) calcd for $[C_{17}H_{10}N_2ClF + Na]^+$ 319.0414, found 319.0408.

3-(3,5-Dimethylphenyl)-5-phenyl-1*H*-pyrrole-2-carbonitrile (**10j**): The title compound was prepared from **6j** (178 mg, 0.65 mmol) and DDQ (171 mg, 0.75 mmol) in 10 mL of toluene according to general procedure B described above. The crude product was purified by column chromatography (ethyl acetate/cyclohexane 1:5) to obtain **10j** (96 mg, 0.35 mmol, 54%) as a pale yellow solid: mp 164–165 °C; R_f 0.24 (ethyl acetate/cyclohexane 1:5); IR (NaCl) $\tilde{\nu}$ 3027, 2917, 2208, 1604, 1485, 1323, 1261, 1032, 849, 760, 690 cm^{-1}; ^1H NMR (400 MHz, $CDCl_3$) δ 9.32 (br s, 1H, NH), 7.60–7.56 (m, 2H, H-2'',6''), 7.48–7.42 (m, 2H, H-2',6'), 7.39–7.33 (m, 3H, H-3'', H-4'', H5''), 7.03–7.00 (m, 1H, H-4'), 6.76 (d, J = 2.9 Hz, 1H, H-4), 2.39 (s, 6H, CH_3) ppm; APT NMR (101 MHz, $CDCl_3$) δ 138.6, 137.7, 137.1, 132.5, 130.6, 129.9, 129.4, 128.6, 125.0, 124.8, 115.7 (CN), 106.5 (C4), 98.1 (C2), 21.5 (2 CH_3) ppm; HRMS (FAB) calcd for $[C_{19}H_{16}N_2 + H]^+$ 273.1392 (273.1386), found 273.1392.

References:

(1) Bergner, I.; Wiebe, C.; Meyer, N.; Opatz, T. *J. Org. Chem.* **2009**, *74*, 8243-8253.

(2) Hall, M. J.; McDonnell, S. O.; Killoran, J.; O'Shea, D. F. *J. Org. Chem.* **2005**, *70*, 5571-5578.

(3) Chen, F.; Shen, T.; Cui, Y.; Jiao, N. *Org. Lett.* **2012**, *14*, 4926-4929.

(4) Alberola, A.; Andres, J. M.; Gonzalez, A.; Pedrosa, R.; Vicente, M. *Heterocycles* **1990**, *31*, 1049-1058.

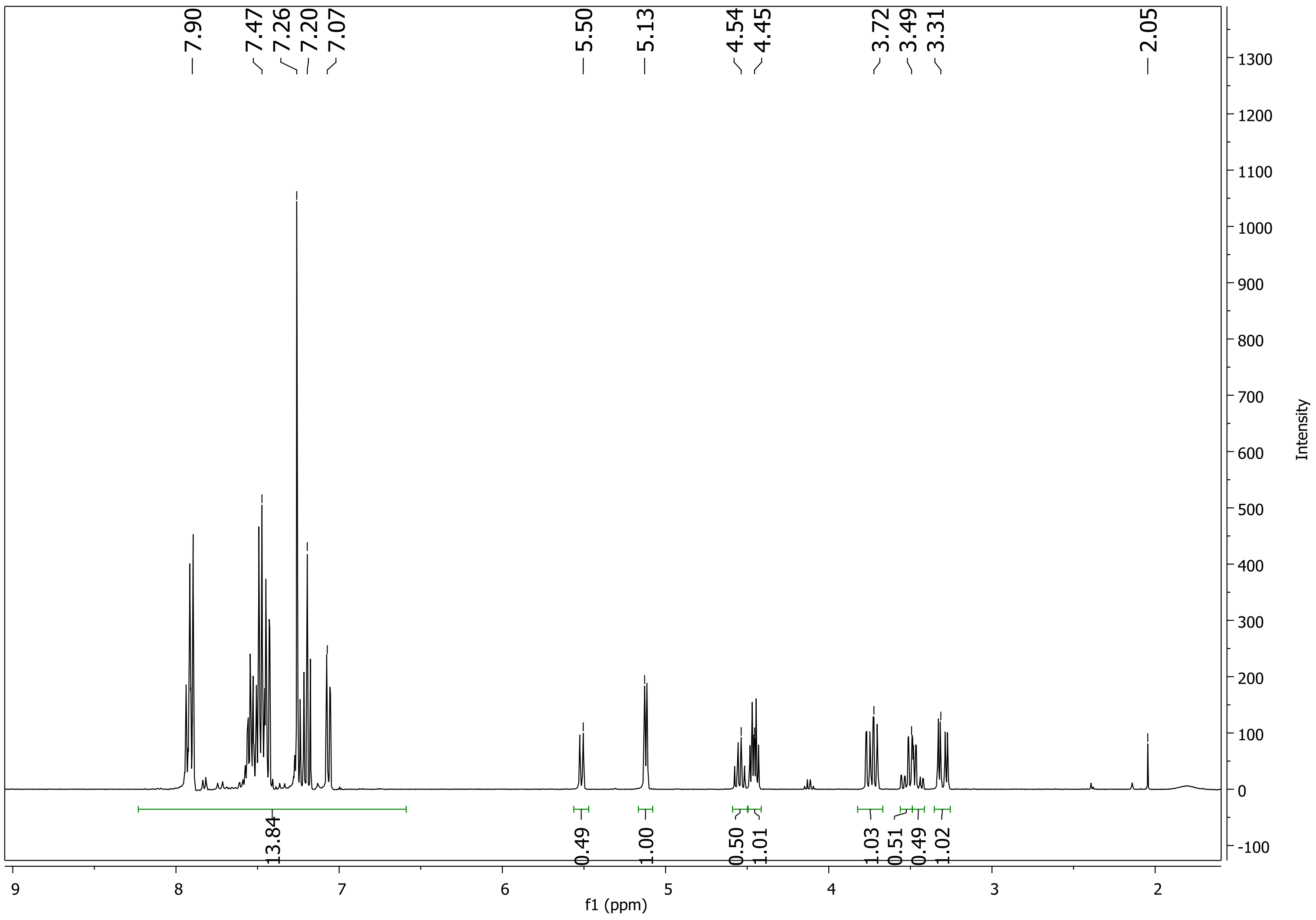

^{1}H NMR (400 MHz, $CDCl_3$) spectrum of **6e** (mixture of isomers)

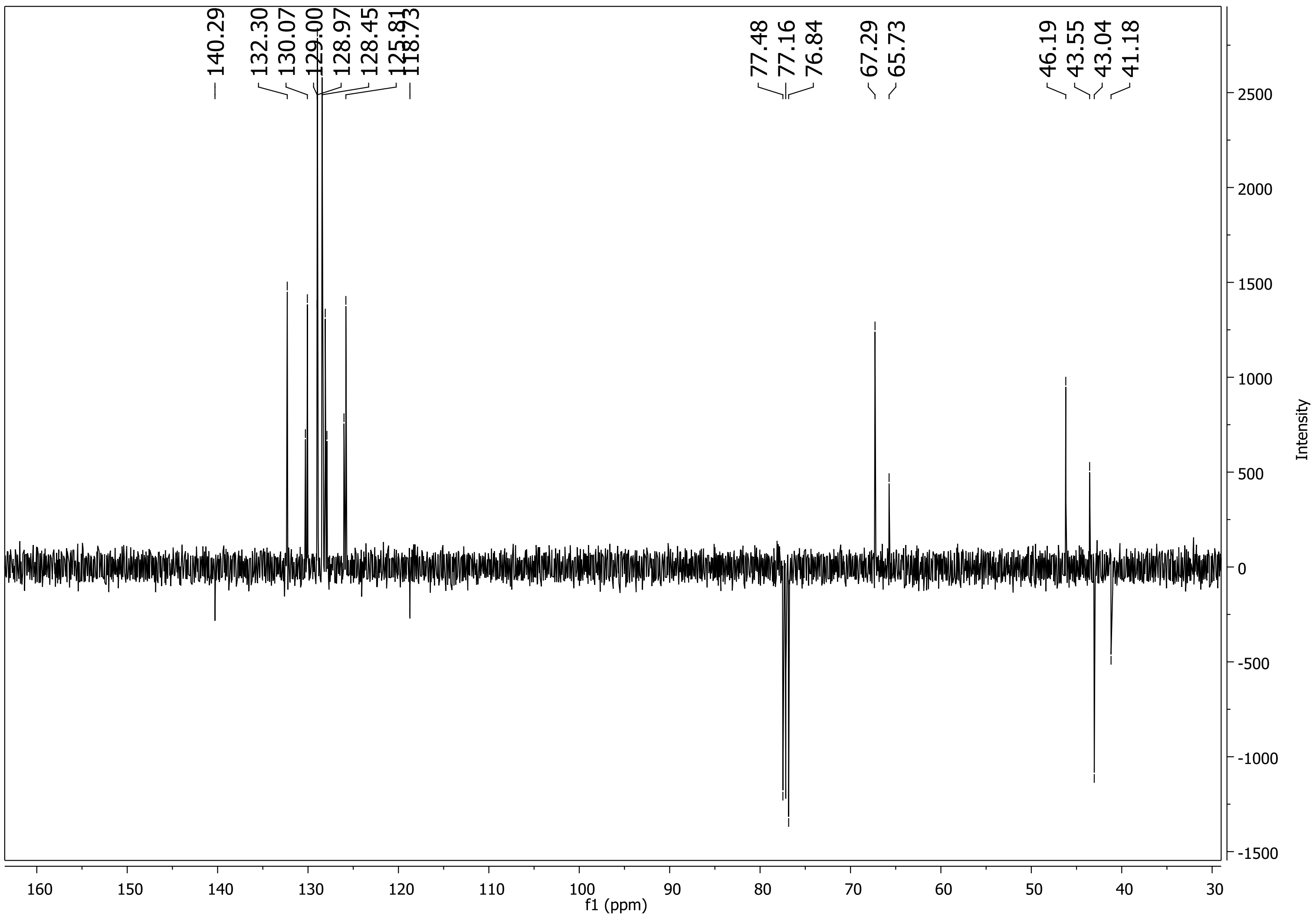

APT NMR (101 MHz, $CDCl_3$) spectrum of **6e** (mixture of isomers)

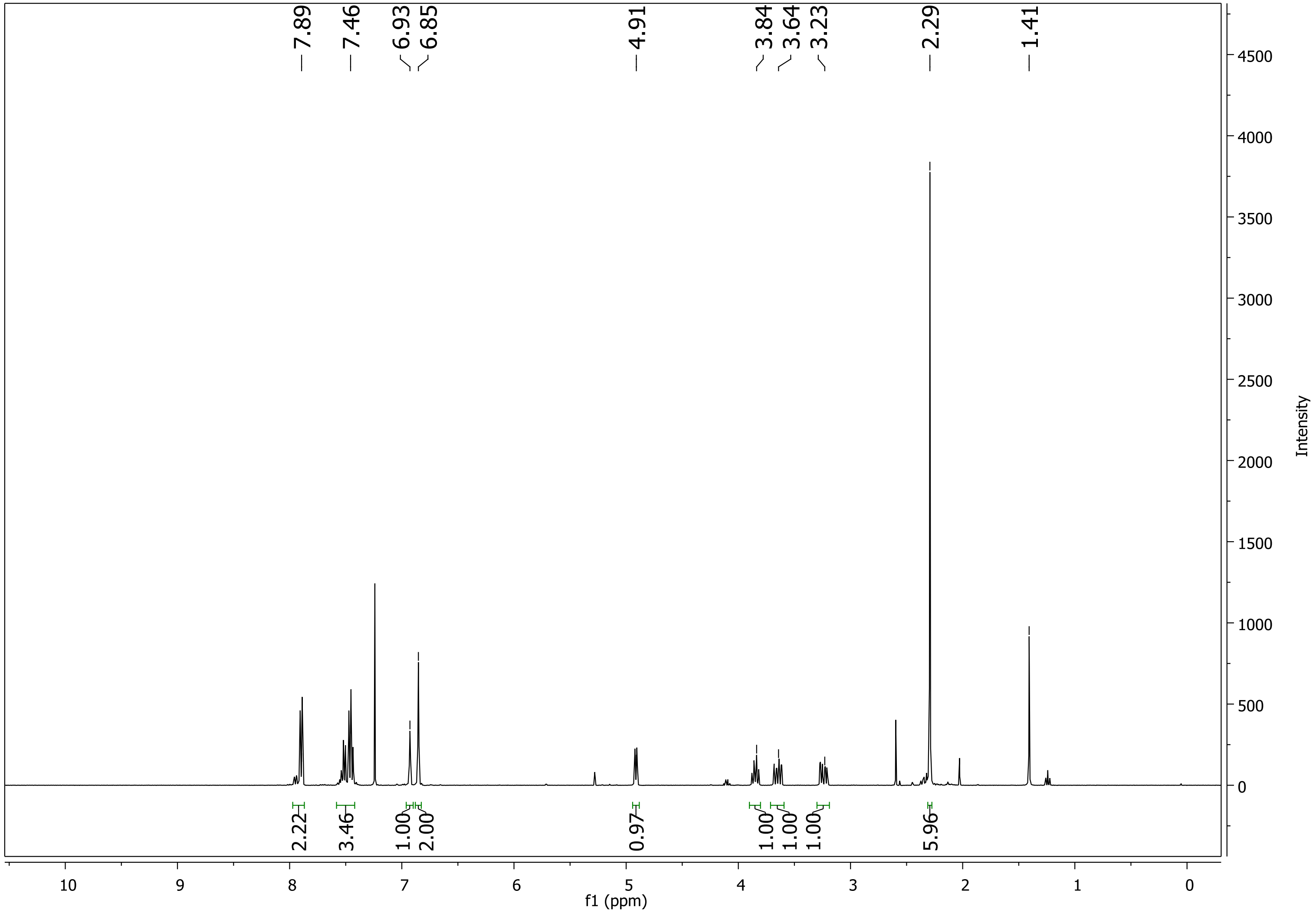

^{1}H NMR (400 MHz, $CDCl_3$) spectrum of *trans*-**6j**

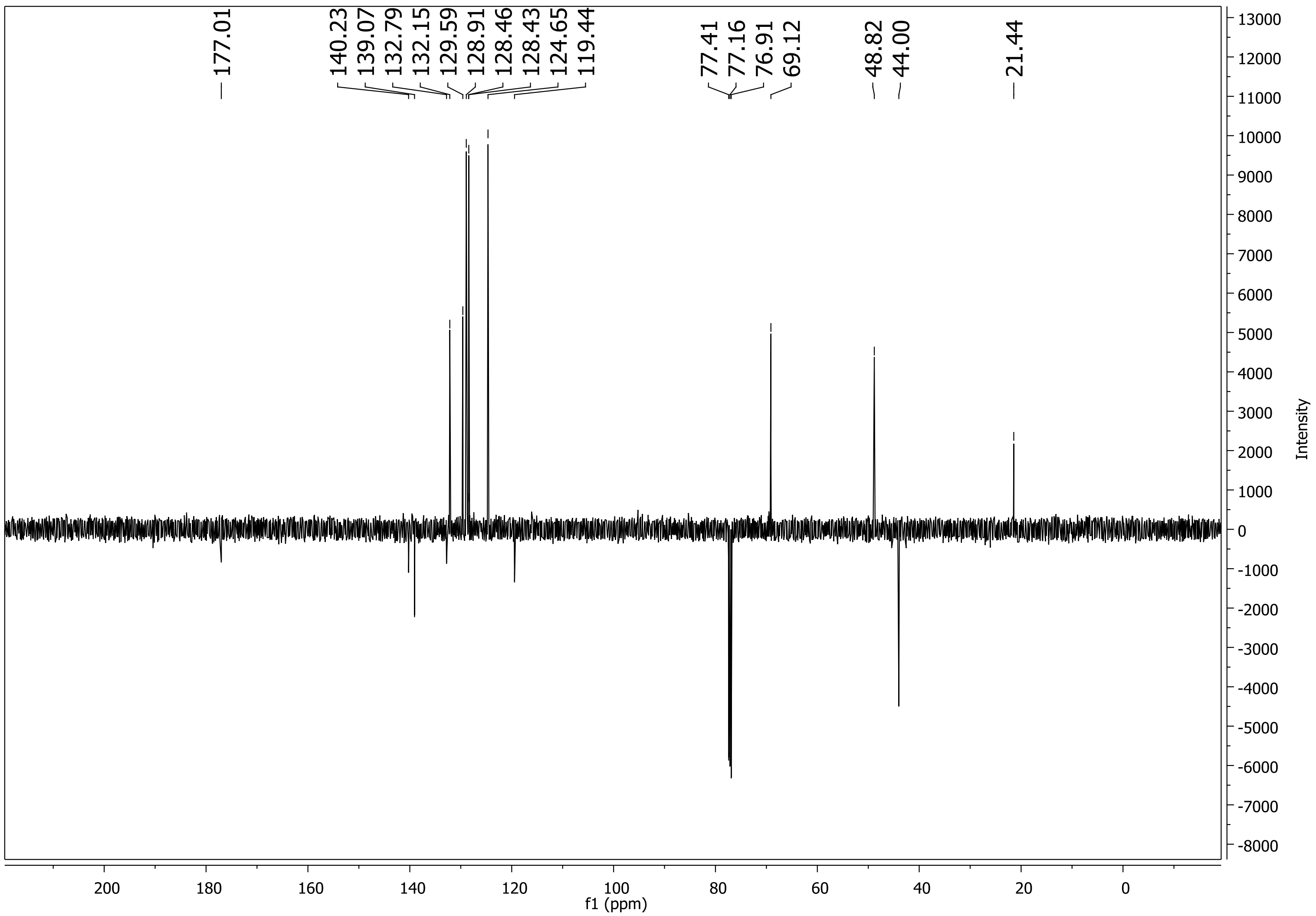

APT NMR (126 MHz, $CDCl_3$) spectrum of *trans*-**6j**

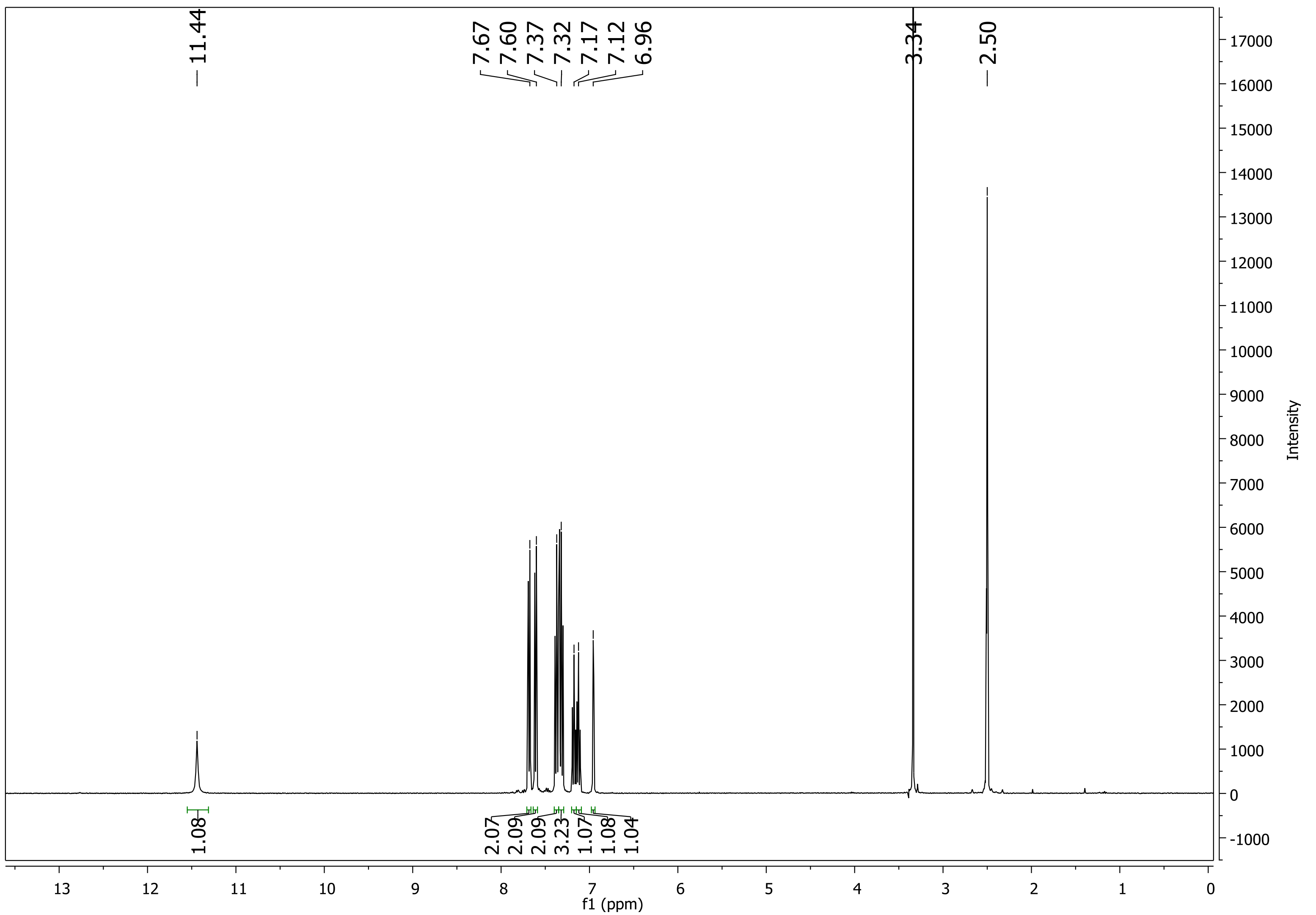

^{1}H NMR (400 MHz, DMSO-d_6) spectrum of **7a**

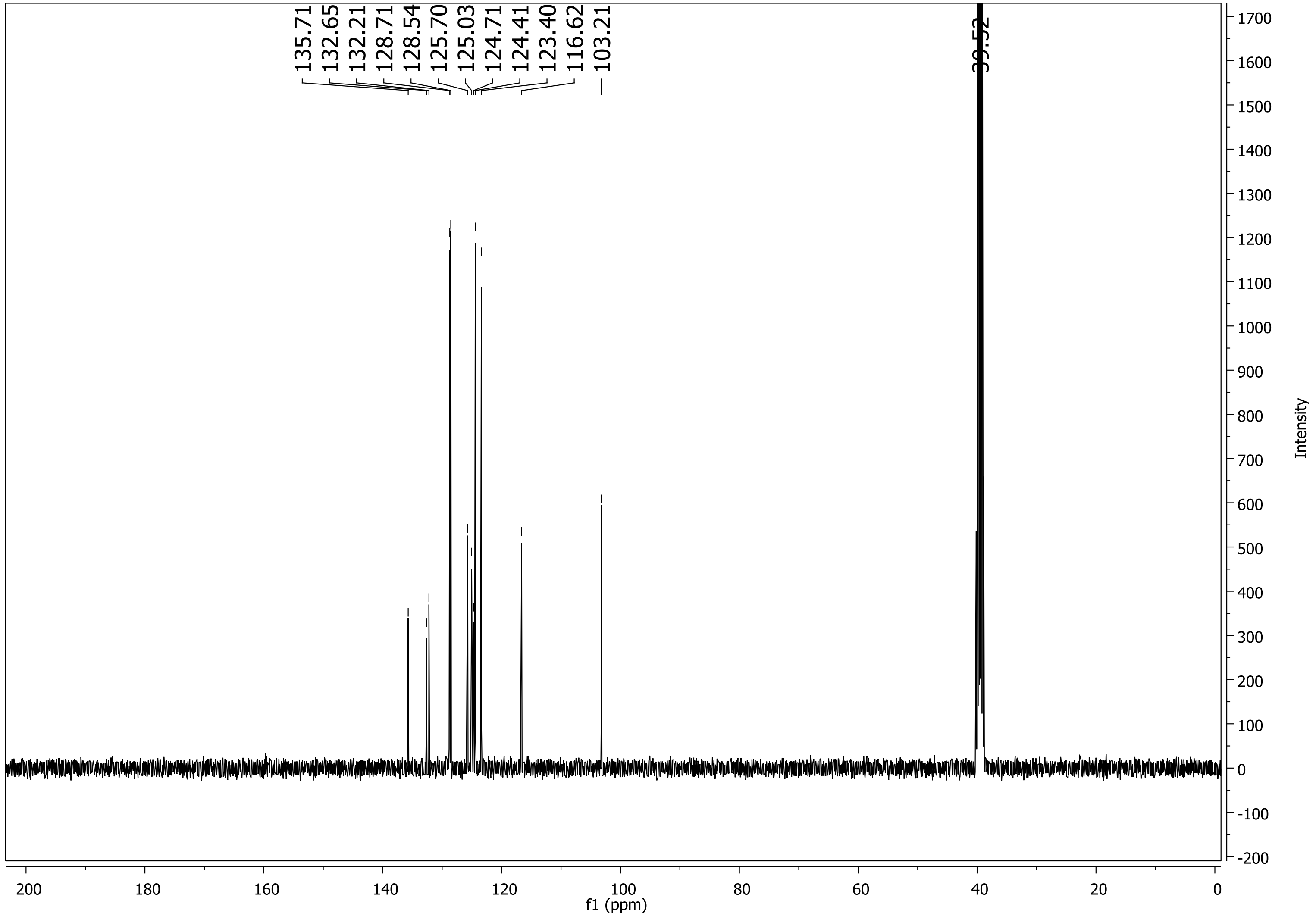

^{13}C NMR (101 MHz, DMSO-d_6) spectrum of **7a**

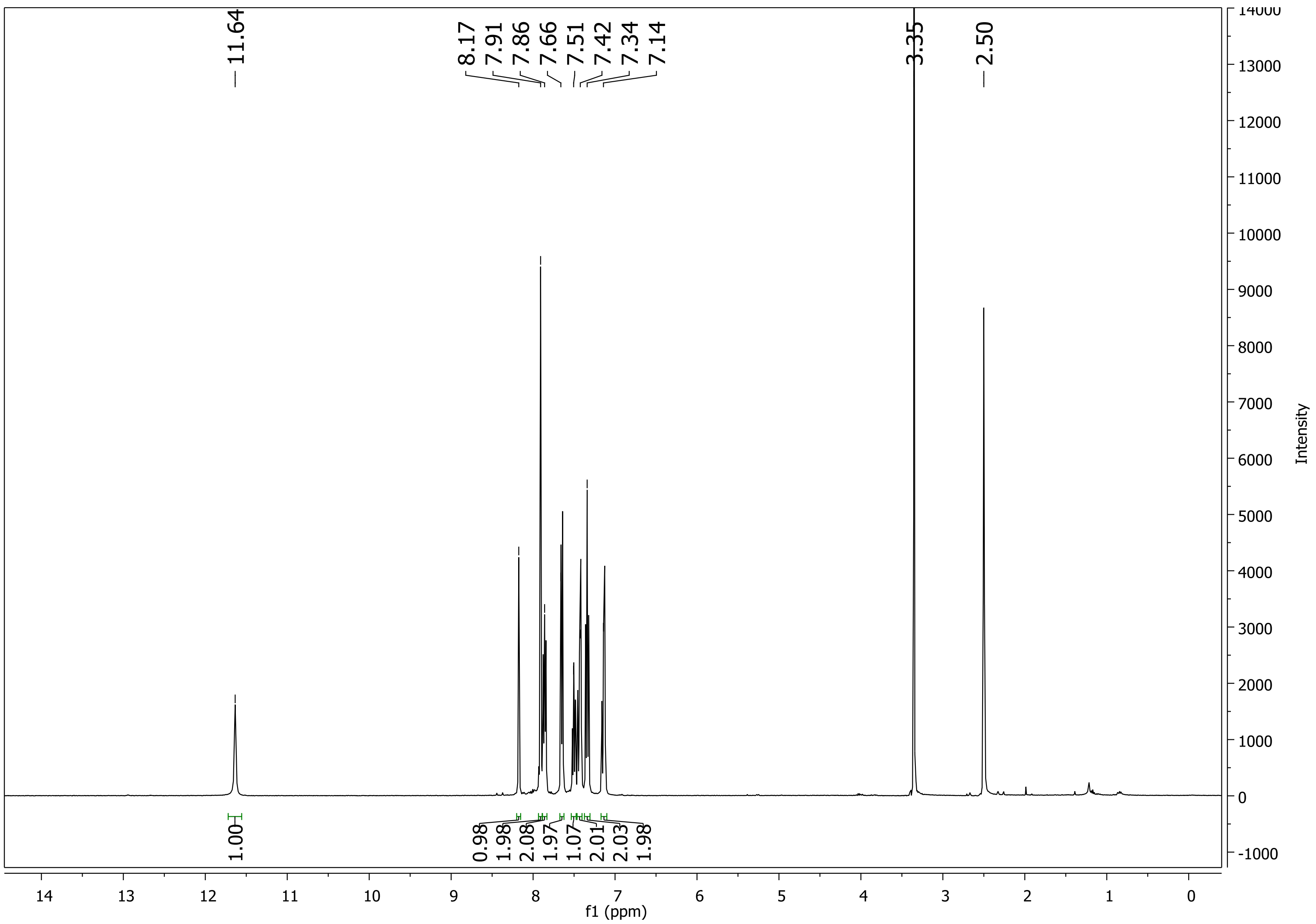

^{1}H NMR (400 MHz, DMSO-d_6) spectrum of **7b**

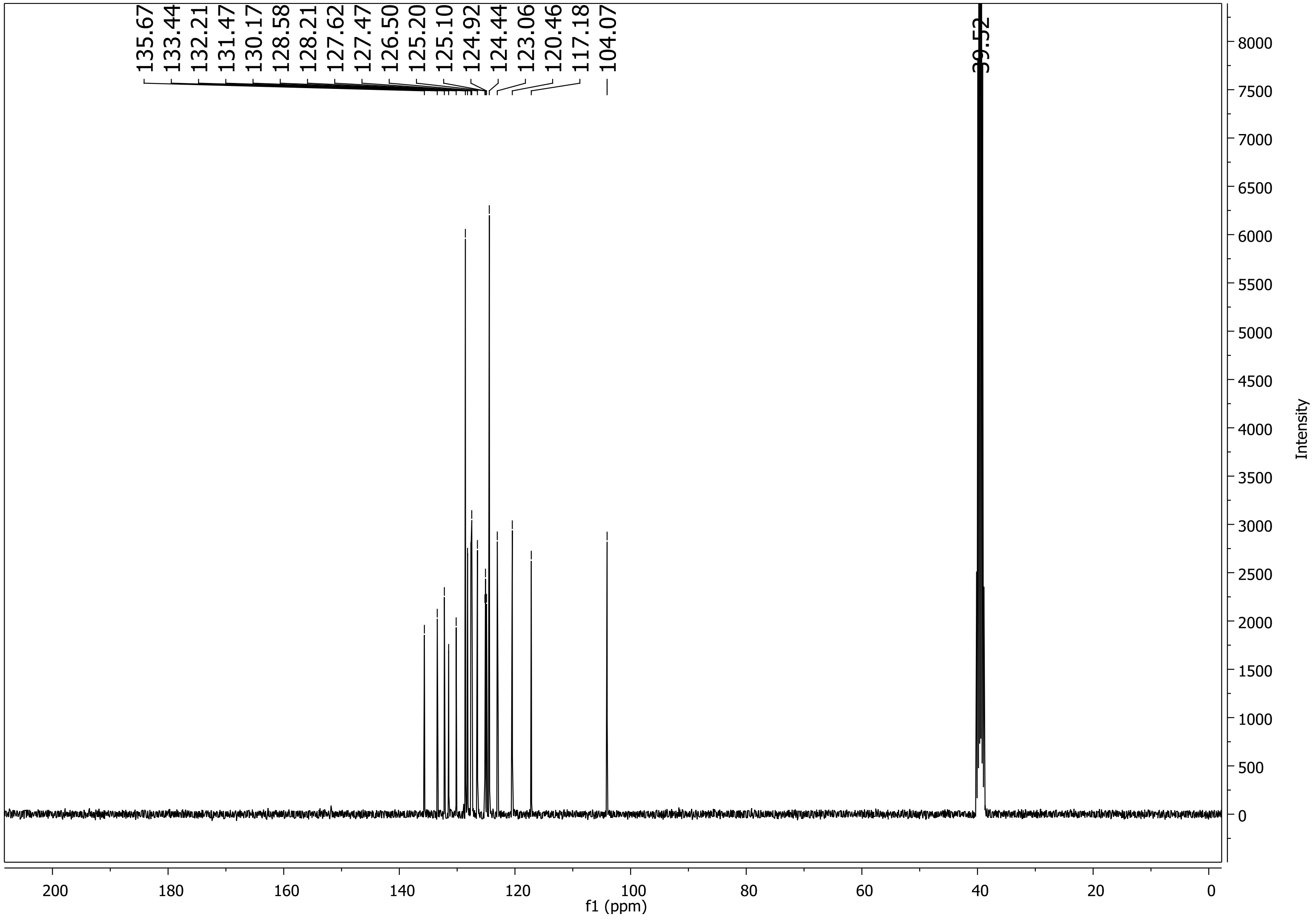

^{13}C NMR (101 MHz, DMSO-d_6) spectrum of **7b**

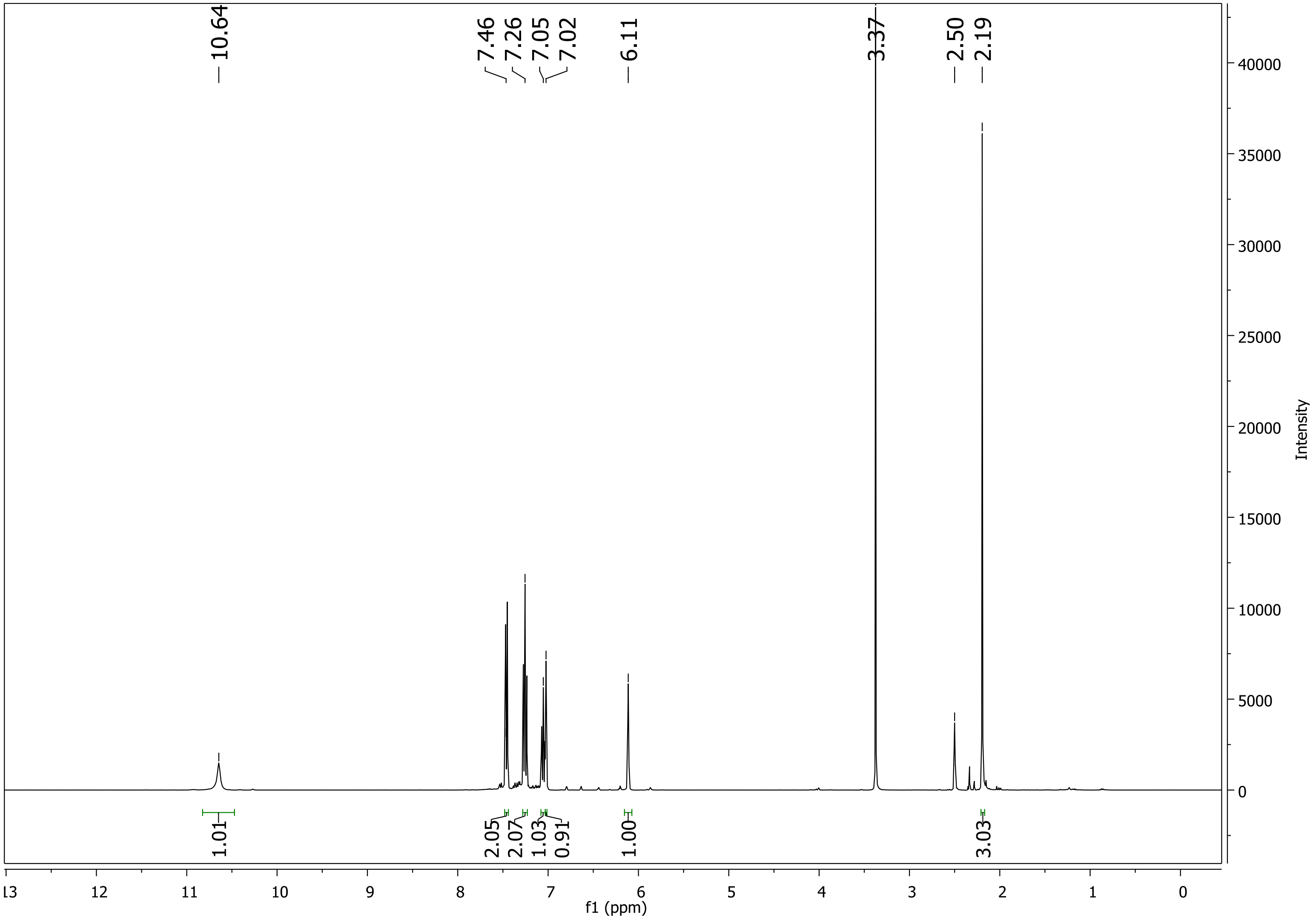

^{1}H NMR (400 MHz, DMSO-d_6) spectrum of **7c**

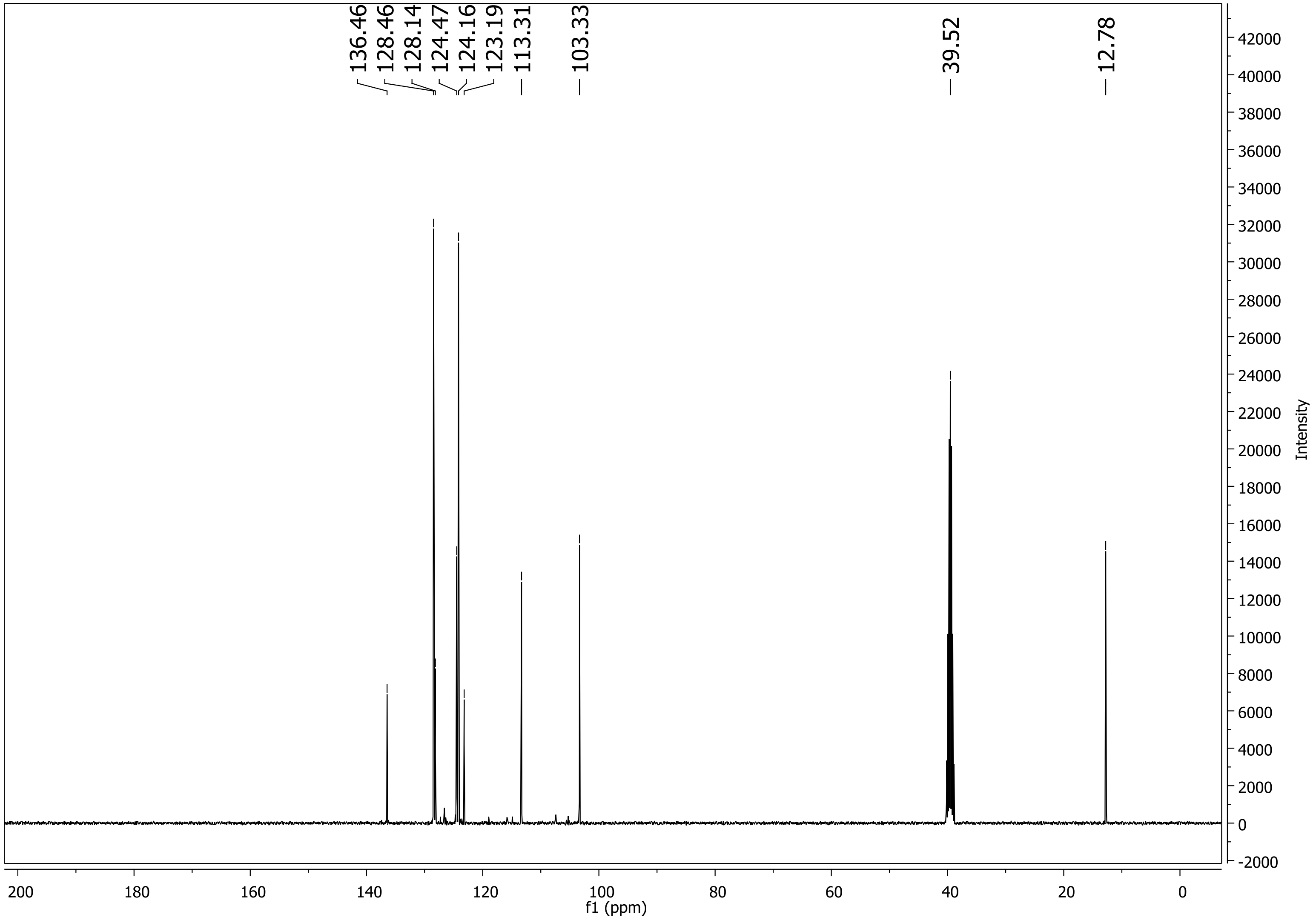

^{13}C NMR (101 MHz, DMSO-d_6) spectrum of **7c**

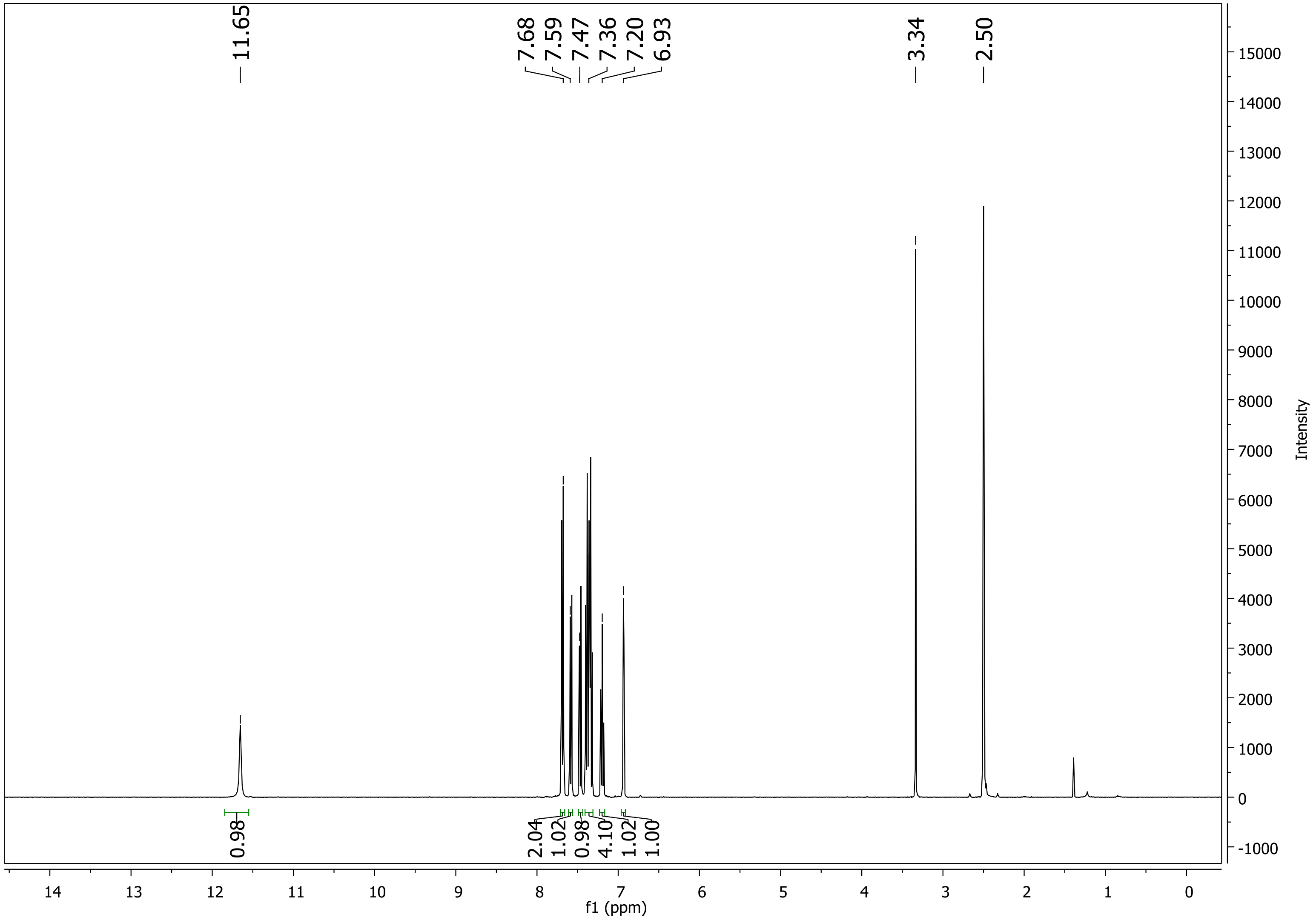

^{1}H NMR (400 MHz, DMSO-d_6) spectrum of **7e**

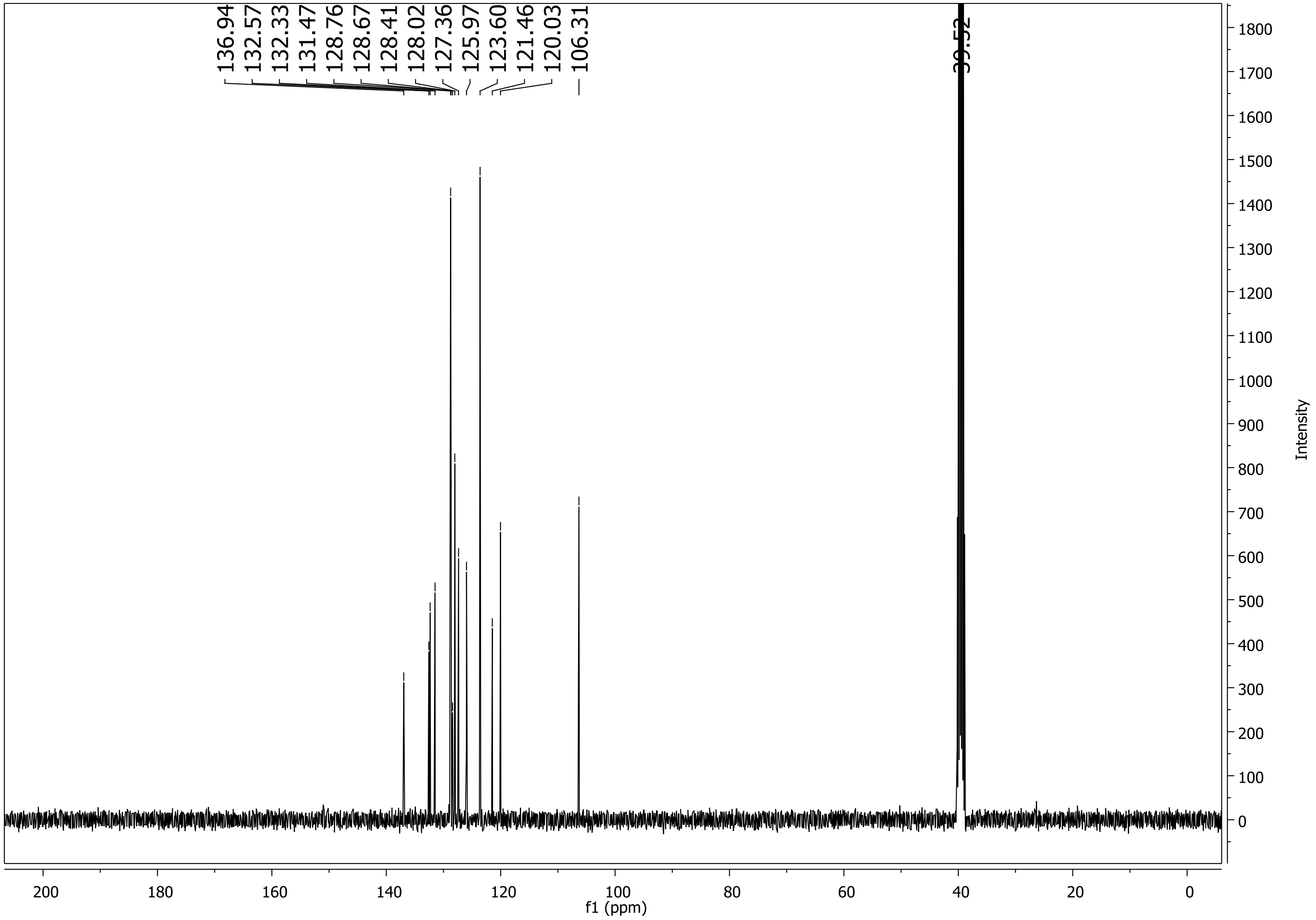

^{13}C NMR (101 MHz, DMSO-d_6) spectrum of **7e**

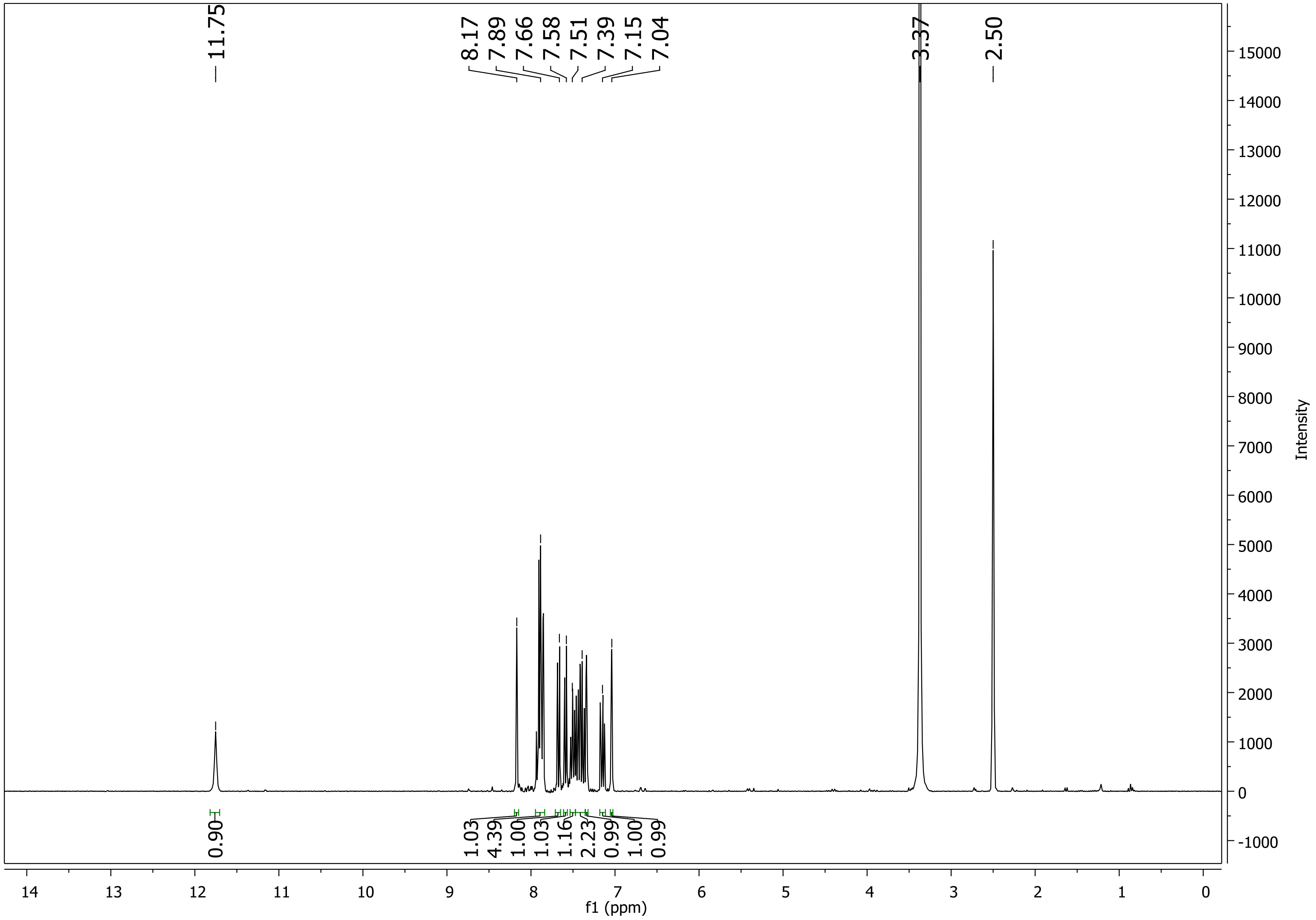

^{1}H NMR (400 MHz, DMSO-d_6) spectrum of **7f**

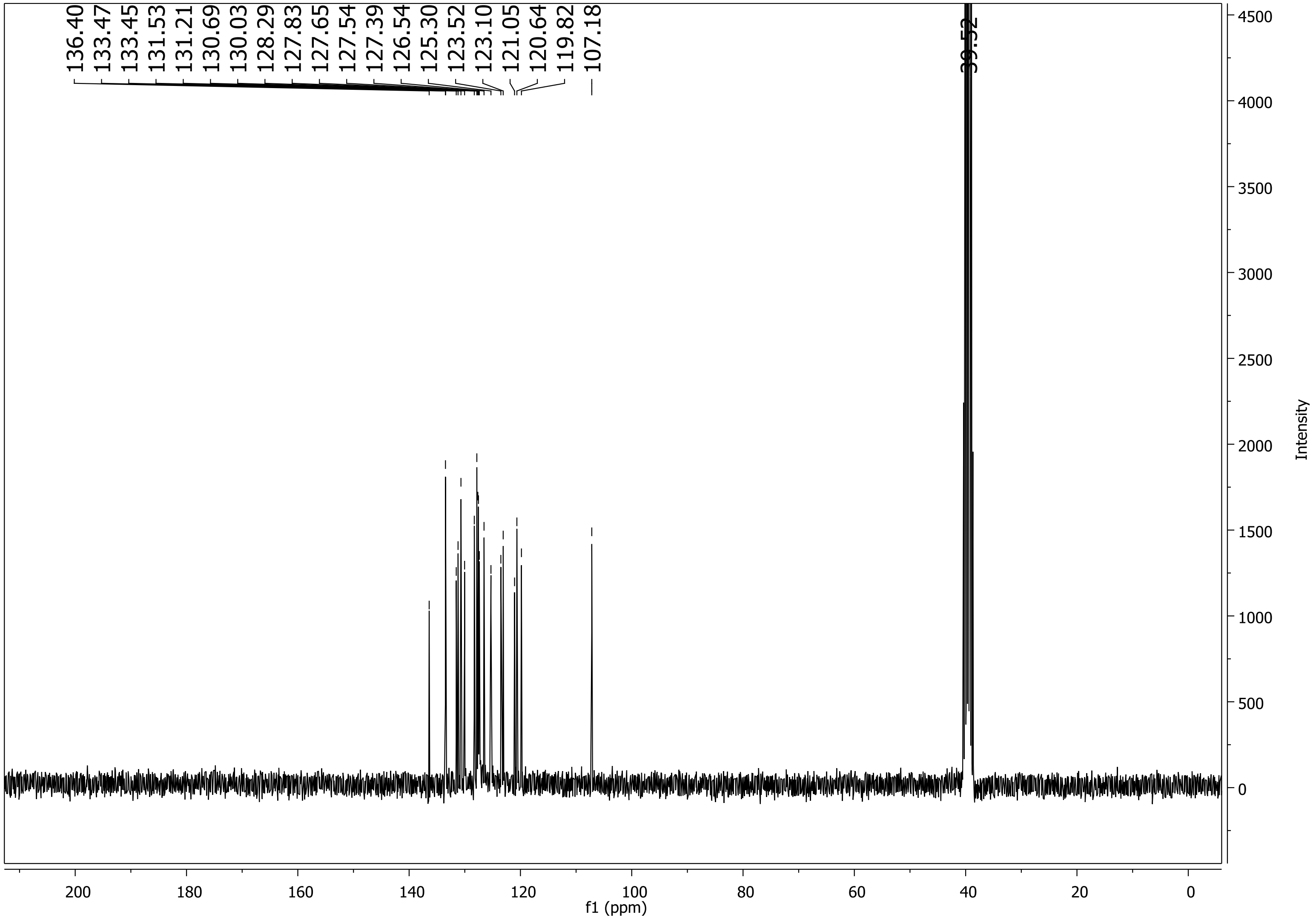

^{13}C NMR (101 MHz, DMSO-d_6) spectrum of **7f**

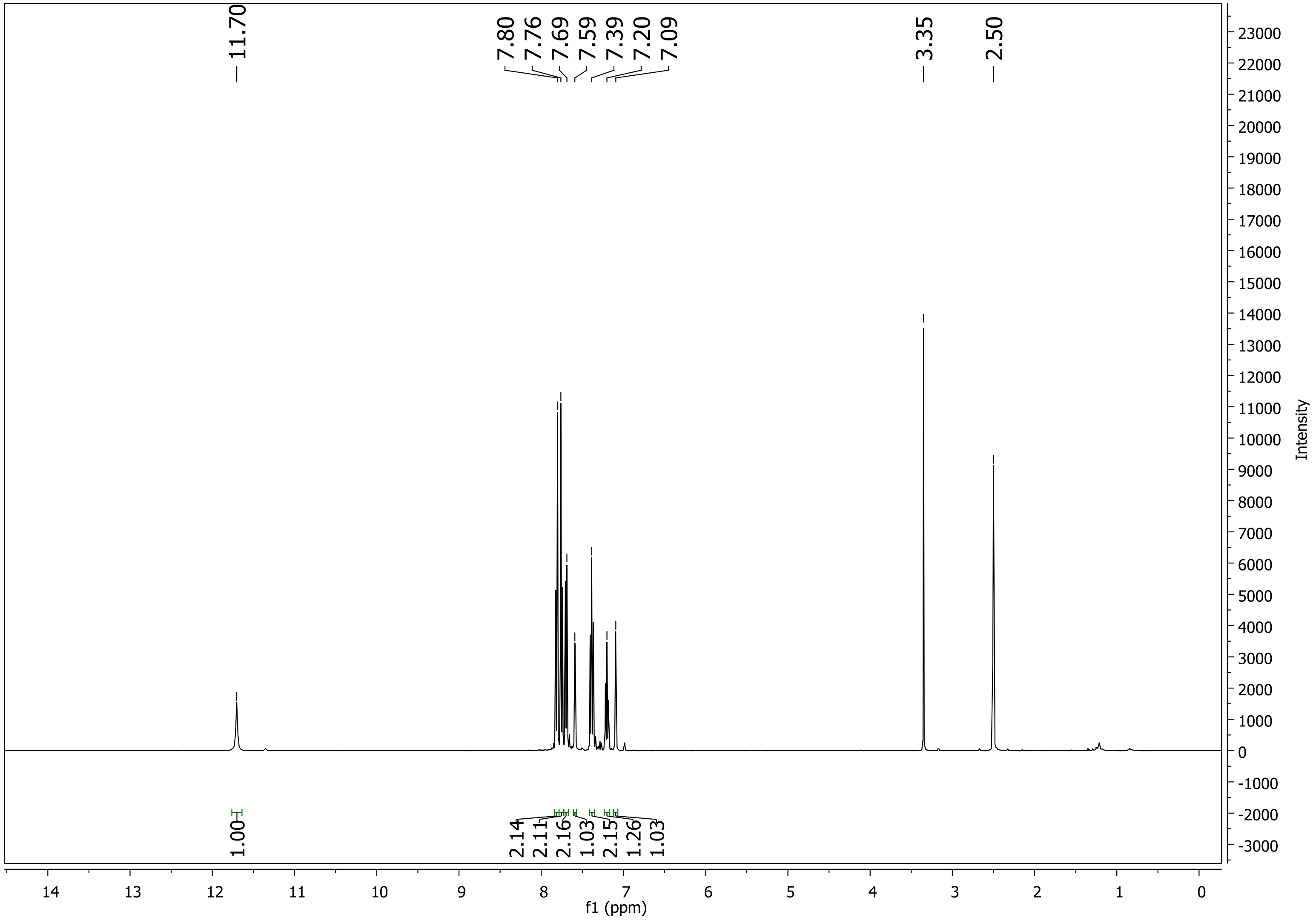

^{1}H NMR (400 MHz, DMSO-d_6) spectrum of **7g**

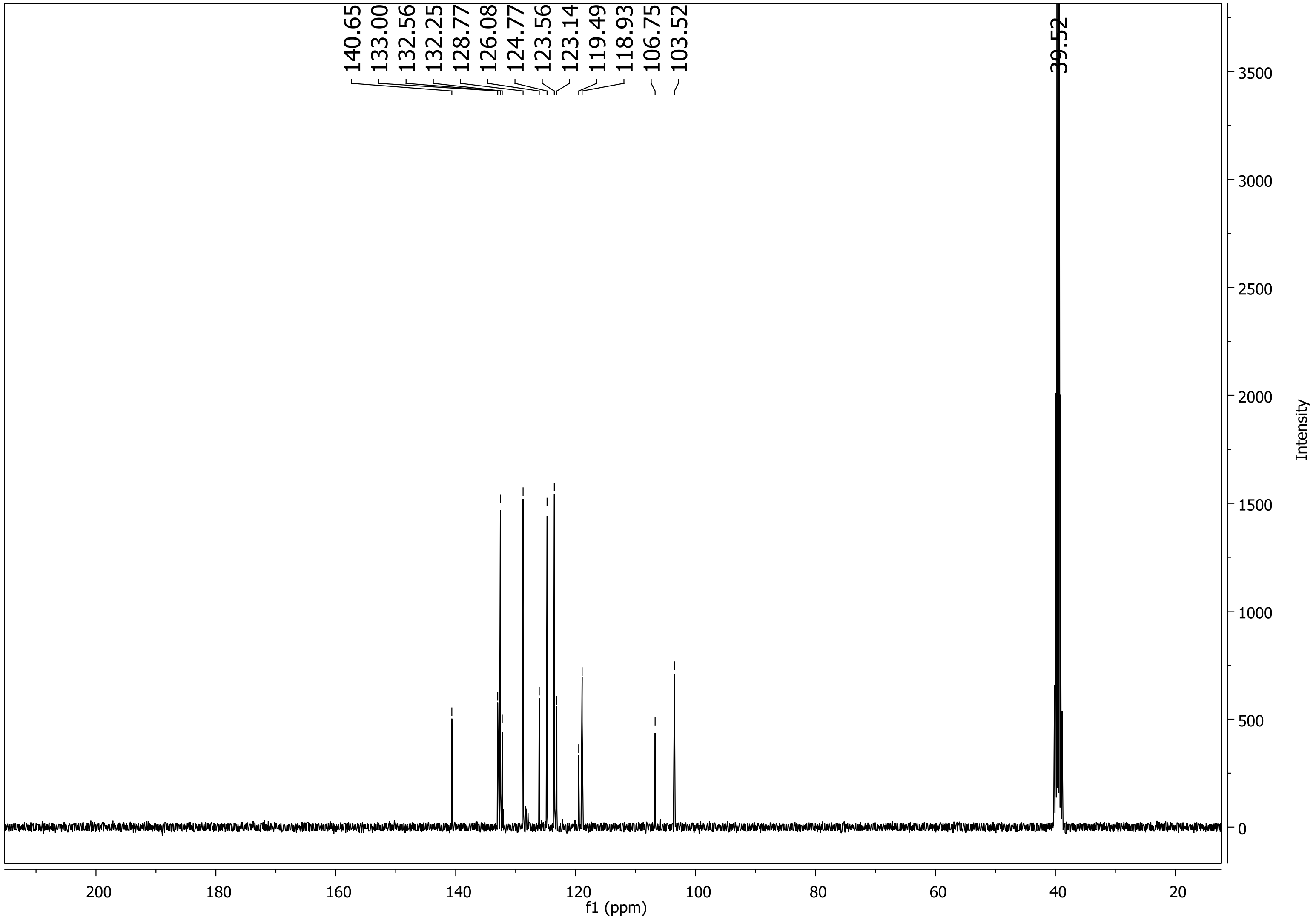

^{13}C NMR (101 MHz, DMSO-d_6) spectrum of **7g**

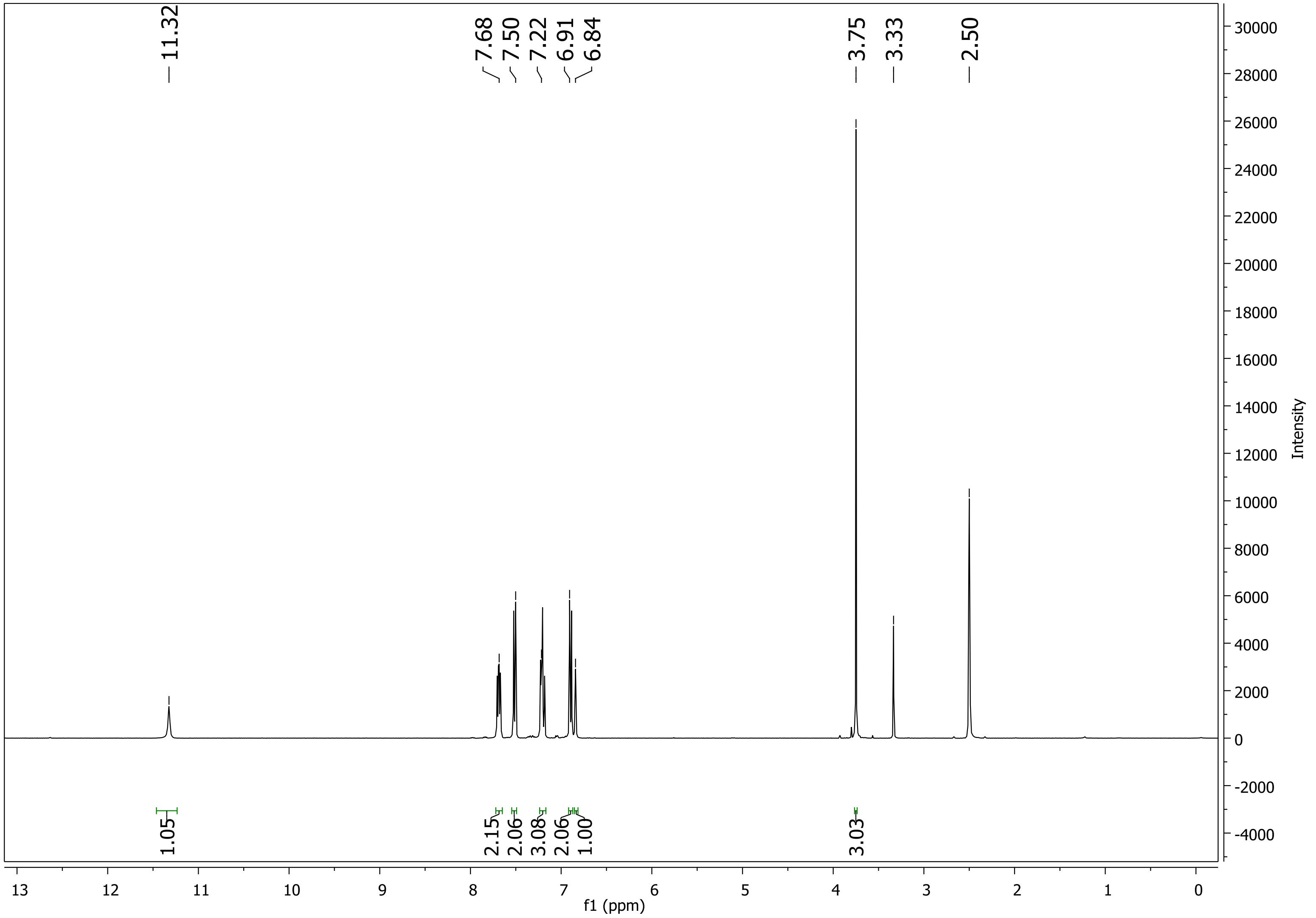

^{1}H NMR (400 MHz, DMSO-d_6) spectrum of **7h**

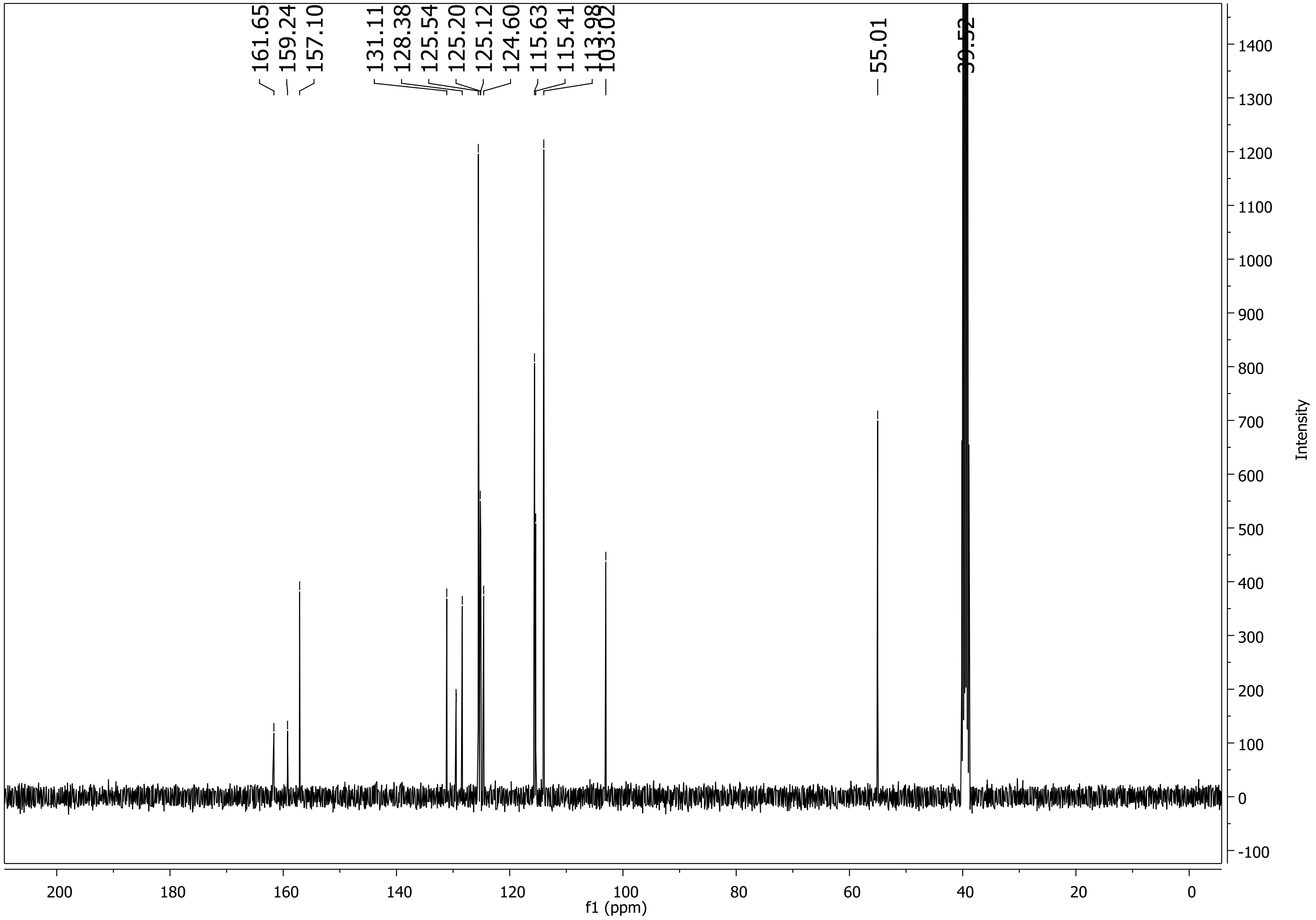

^{13}C NMR (101 MHz, DMSO-d_6) spectrum of **7h**

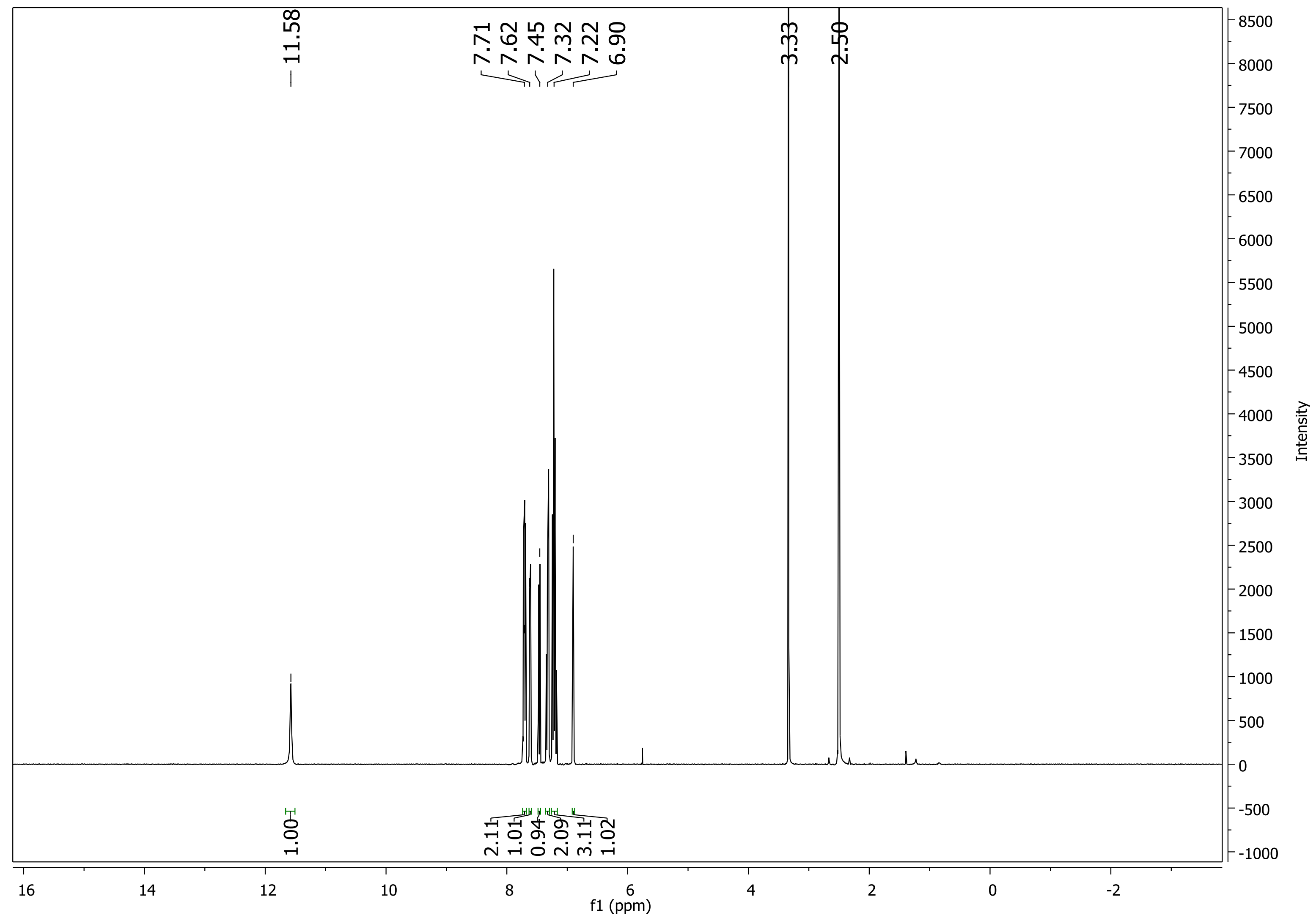

^{1}H NMR (400 MHz, DMSO-d_6) spectrum of **7i**

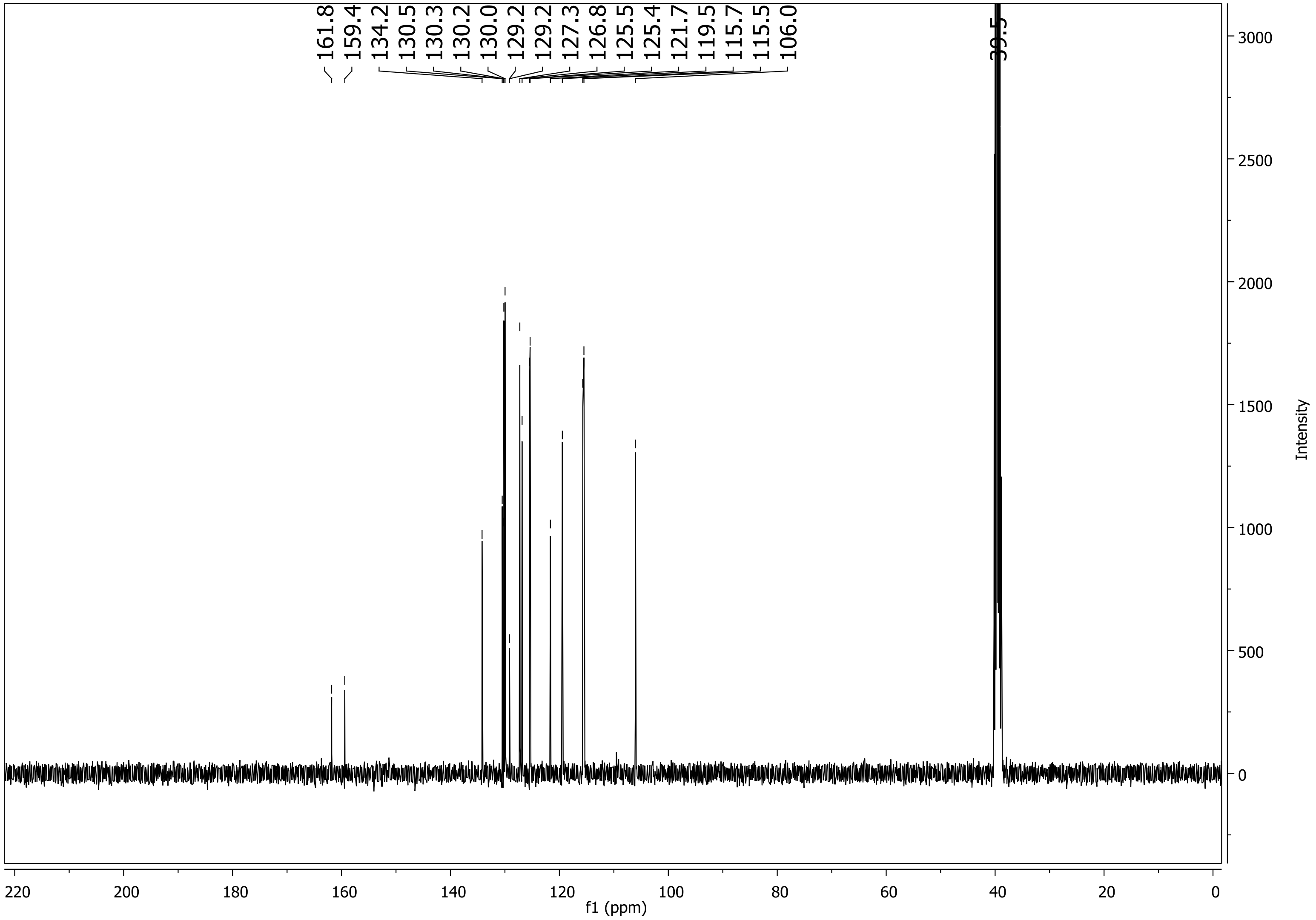

^{13}C NMR (101 MHz, DMSO-d_6) spectrum of **7i**

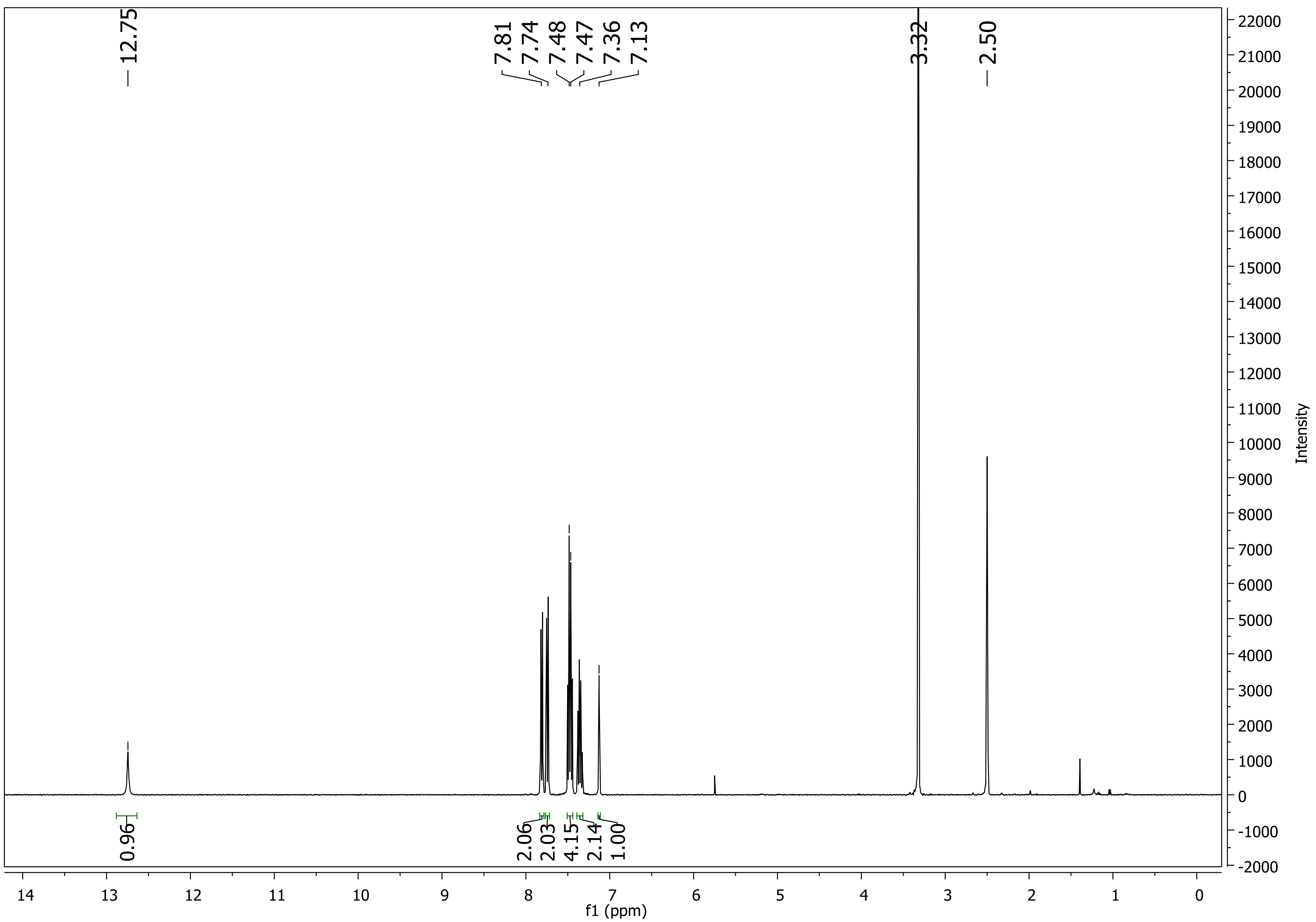

^{1}H NMR (400 MHz, DMSO-d_6) spectrum of **10a**

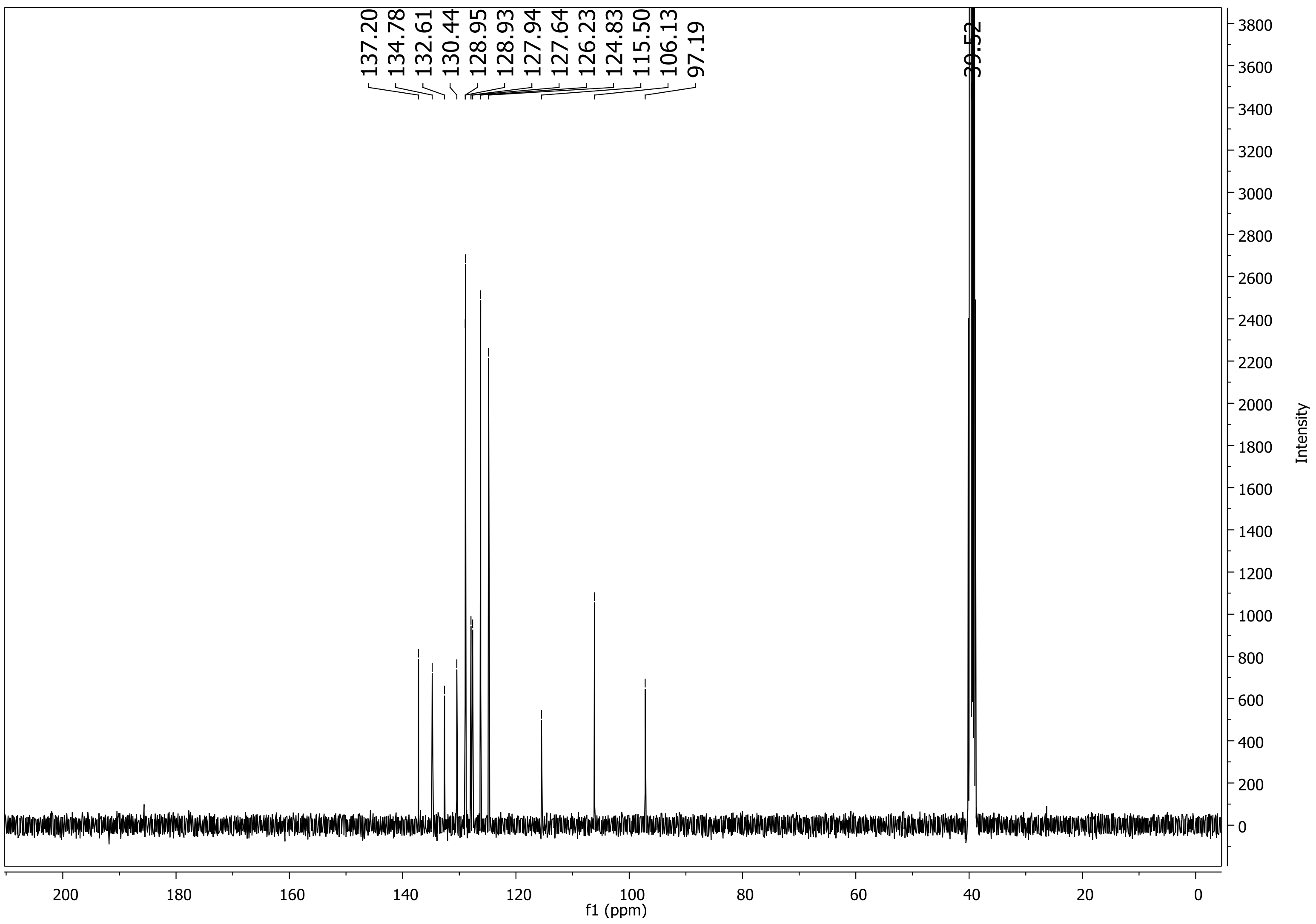

^{13}C NMR (101 MHz, DMSO-d_6) spectrum of **10a**

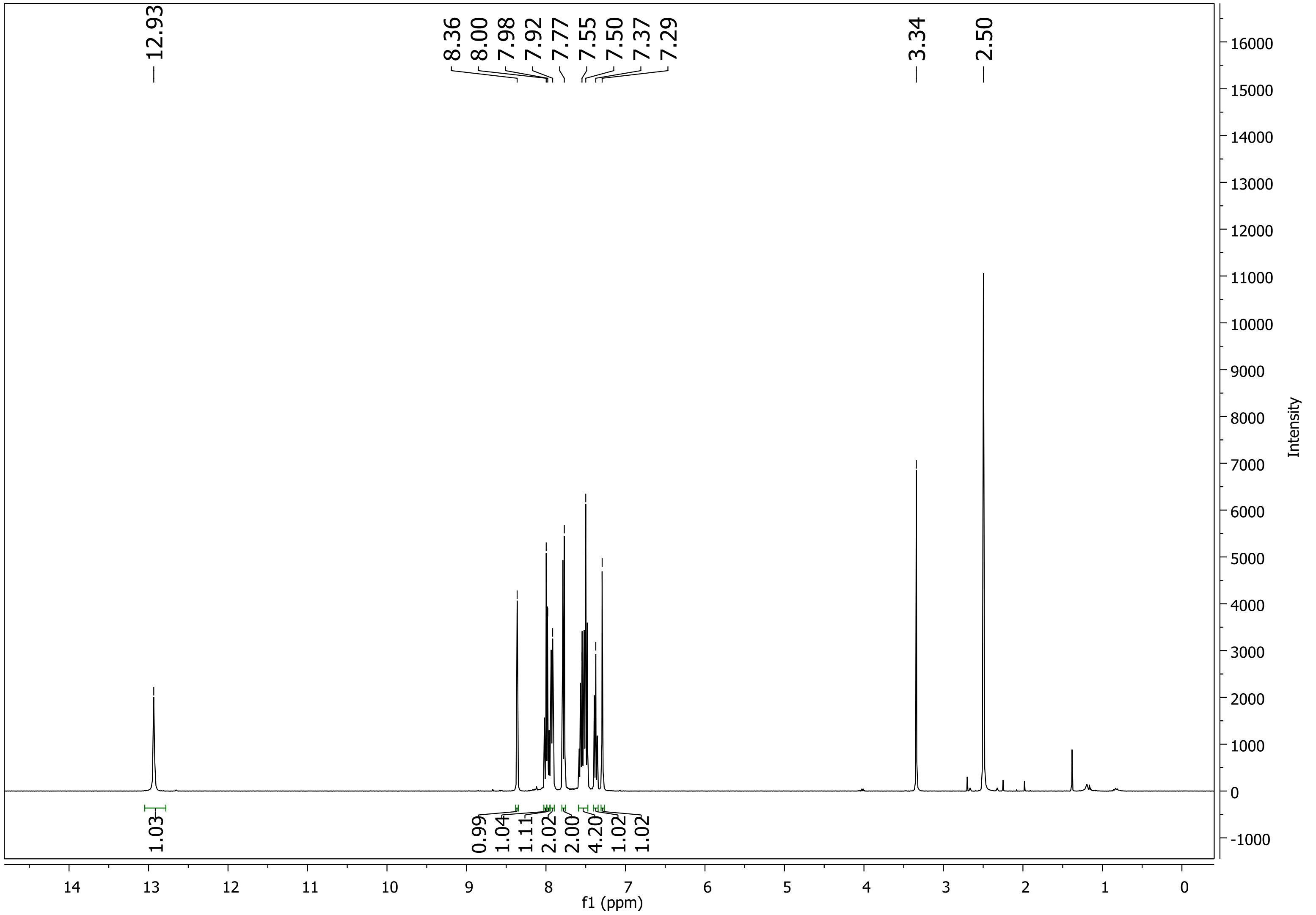

^{1}H NMR (400 MHz, DMSO-d_6) spectrum of **10b**

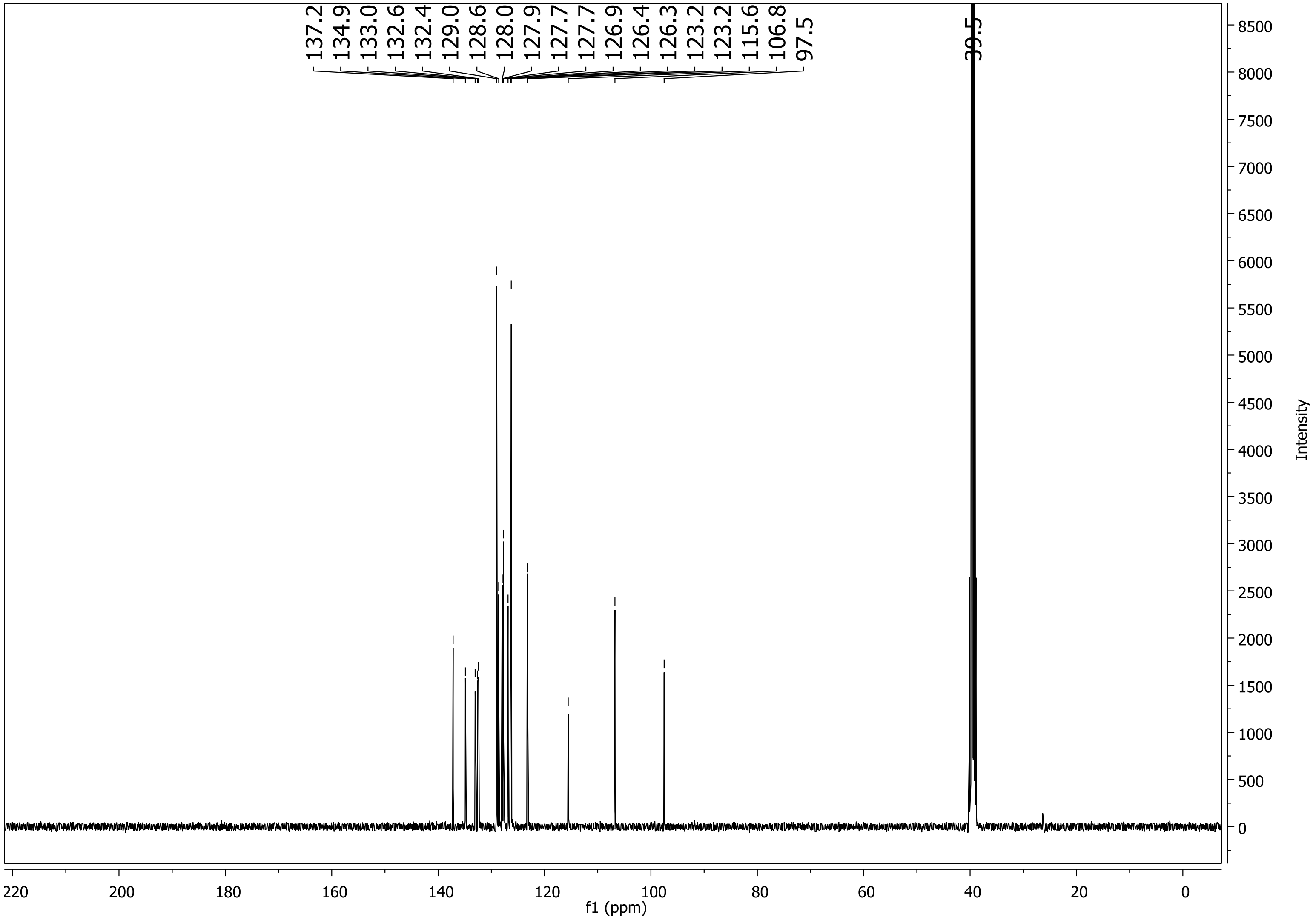

^{13}C NMR (101 MHz, DMSO-d_6) spectrum of **10b**

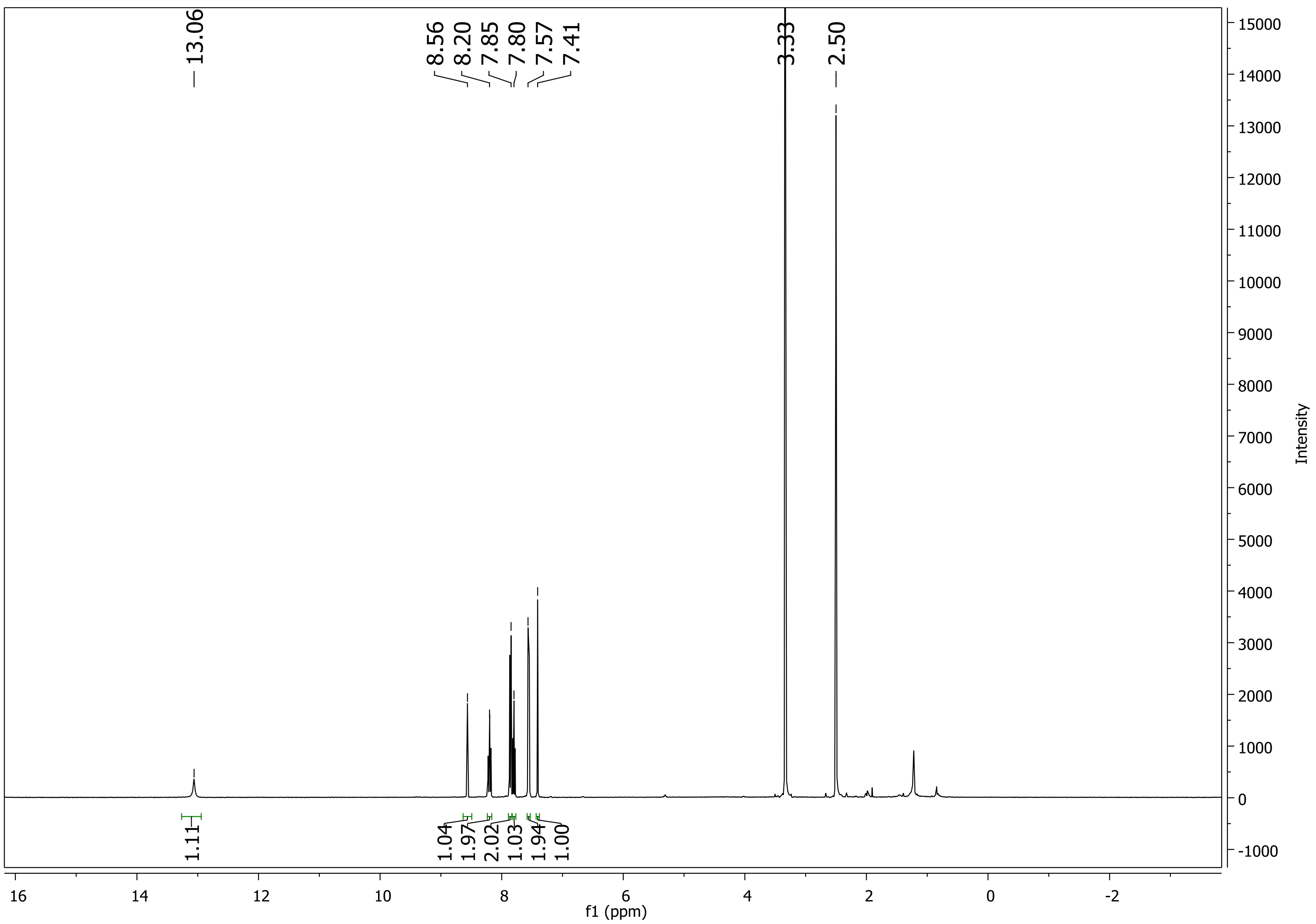

^{1}H NMR (400 MHz, DMSO-d_6) spectrum of **10d**

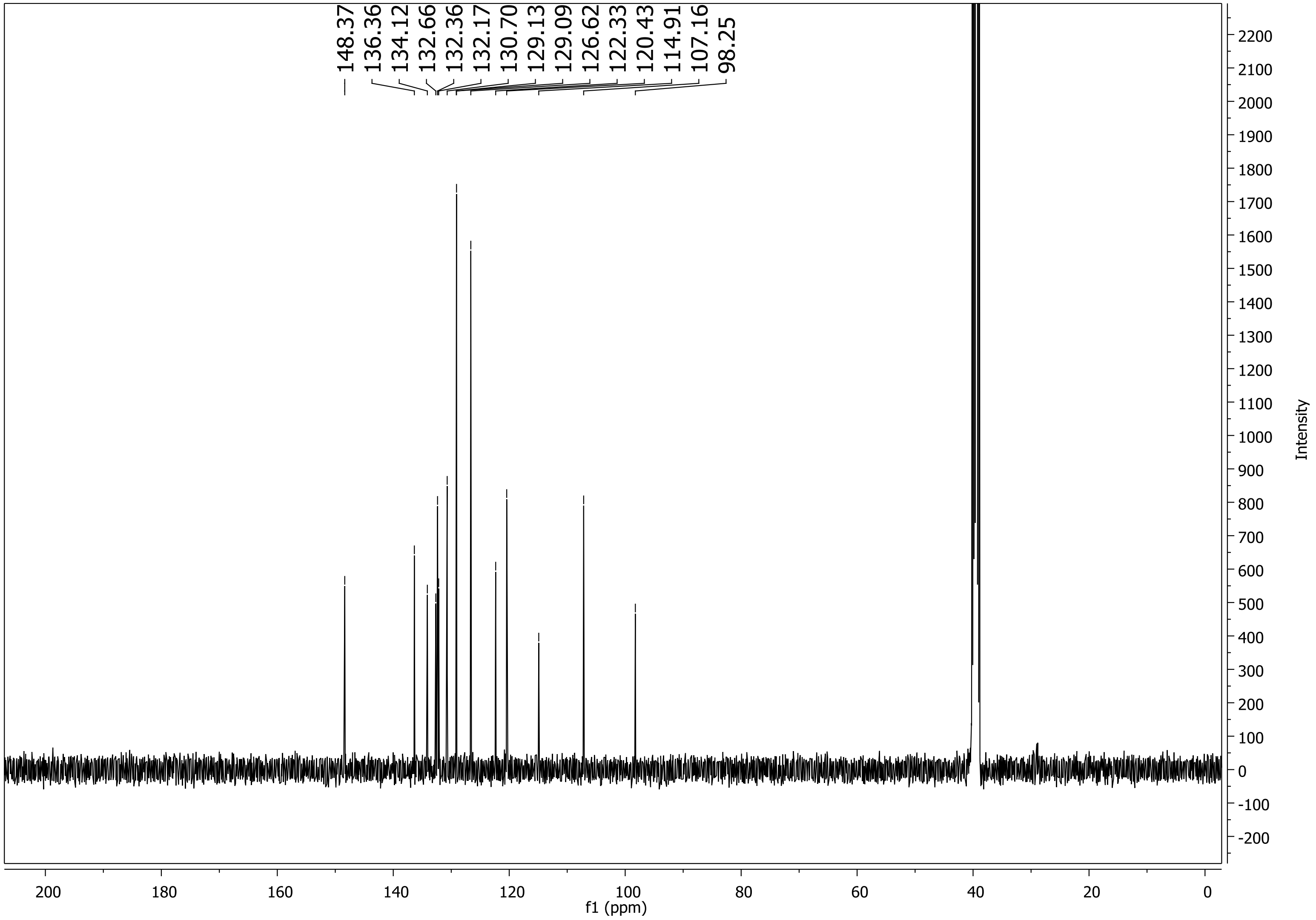

^{13}C NMR (101 MHz, DMSO-d_6) spectrum of **10d**

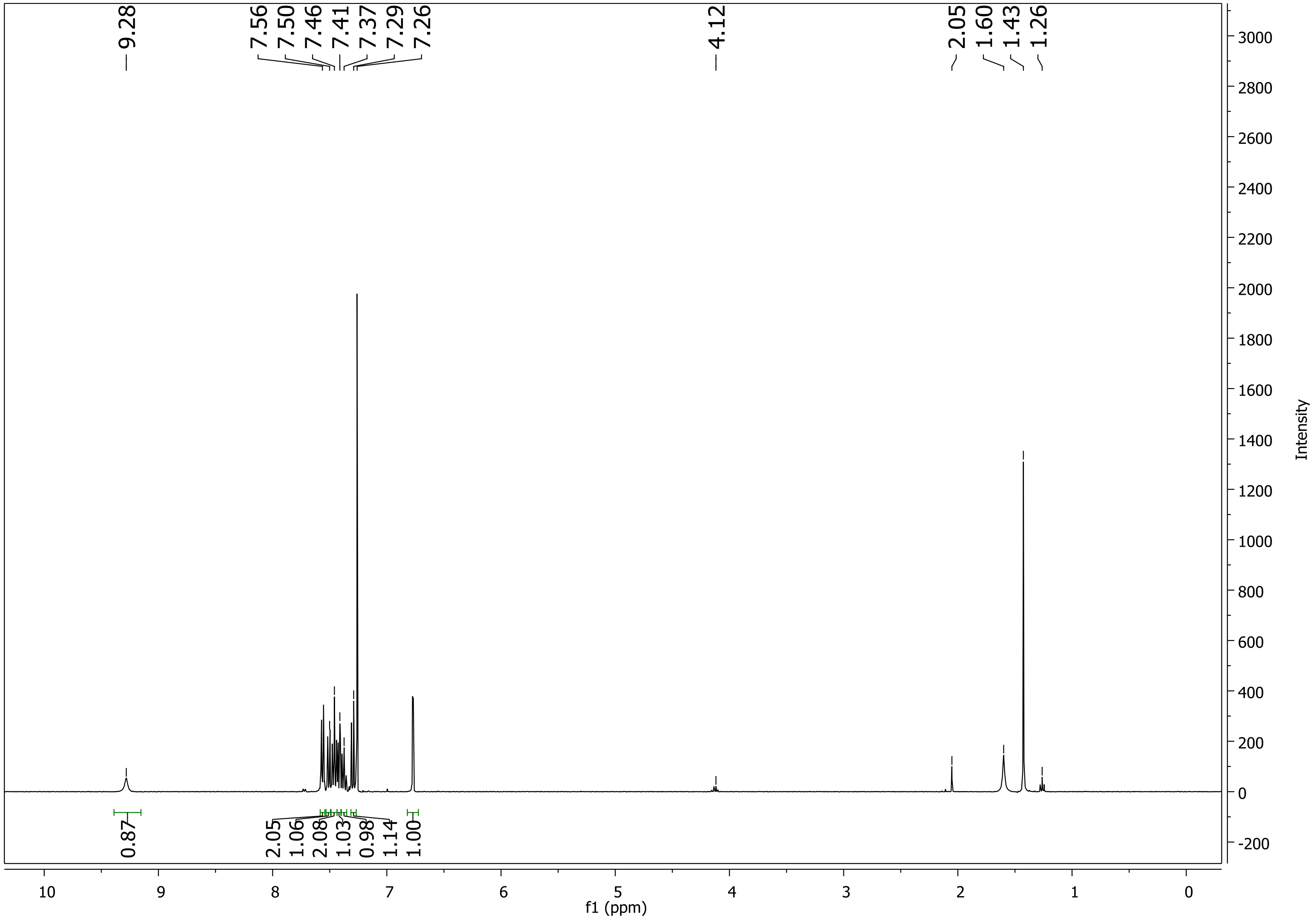

^{1}H NMR (400 MHz, $CDCl_3$) spectrum of **10e**

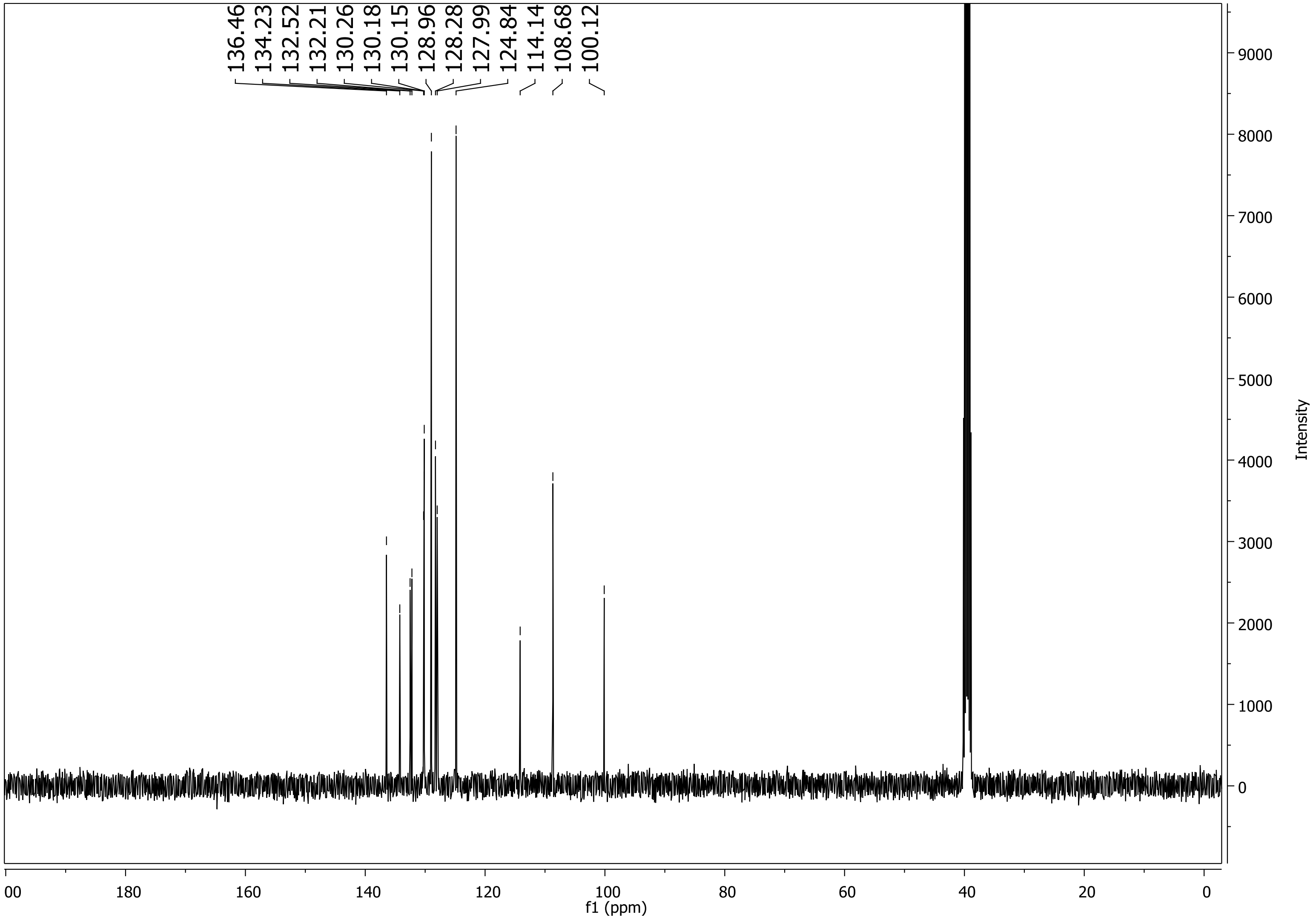

^{13}C NMR (101 MHz, DMSO-d_6) spectrum of **10e**

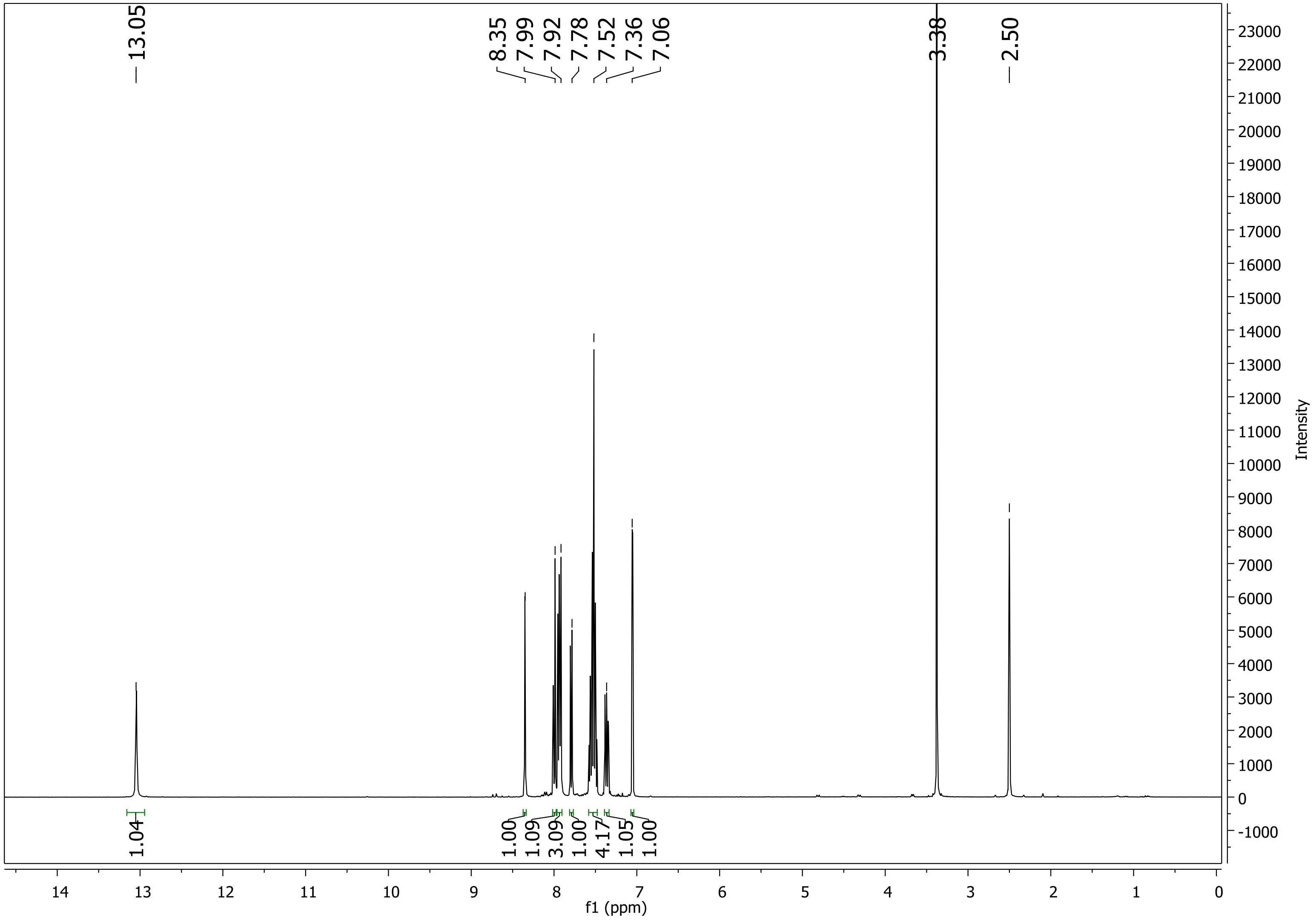

^{1}H NMR (400 MHz, DMSO-d_6) spectrum of **10f**

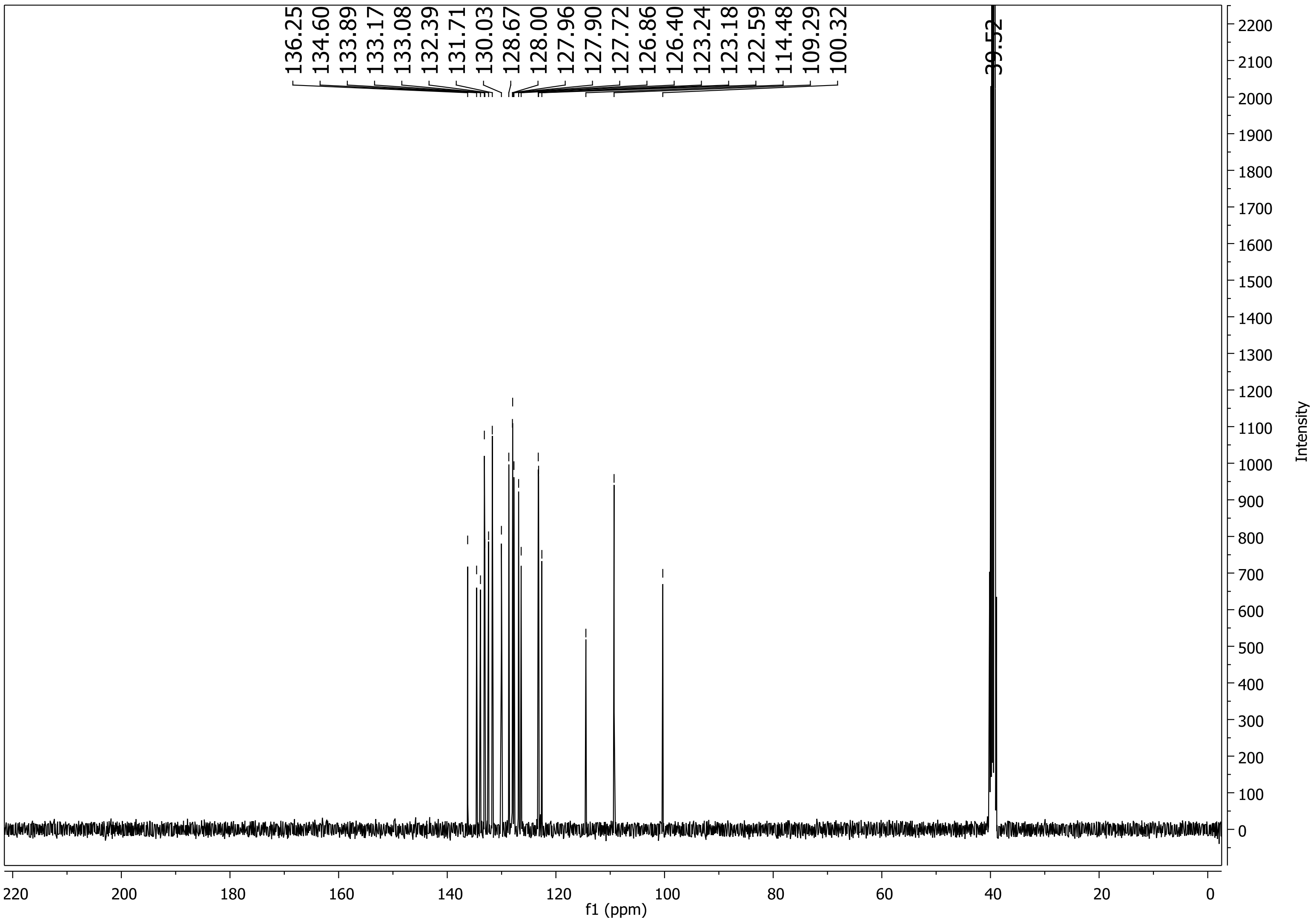

^{13}C NMR (101 MHz, DMSO-d_6) spectrum of **10f**

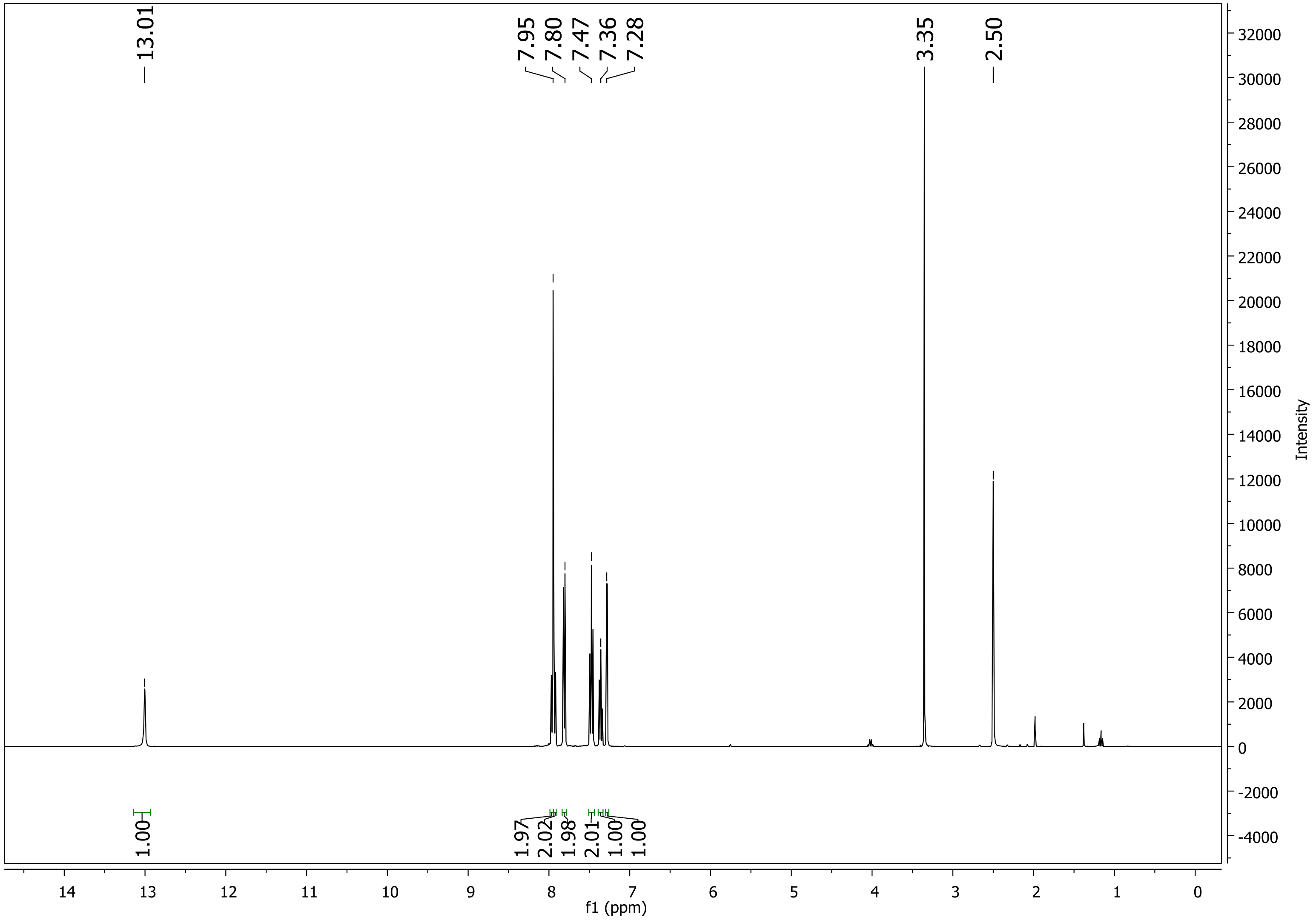

^{1}H NMR (400 MHz, DMSO-d_6) spectrum of **10g**

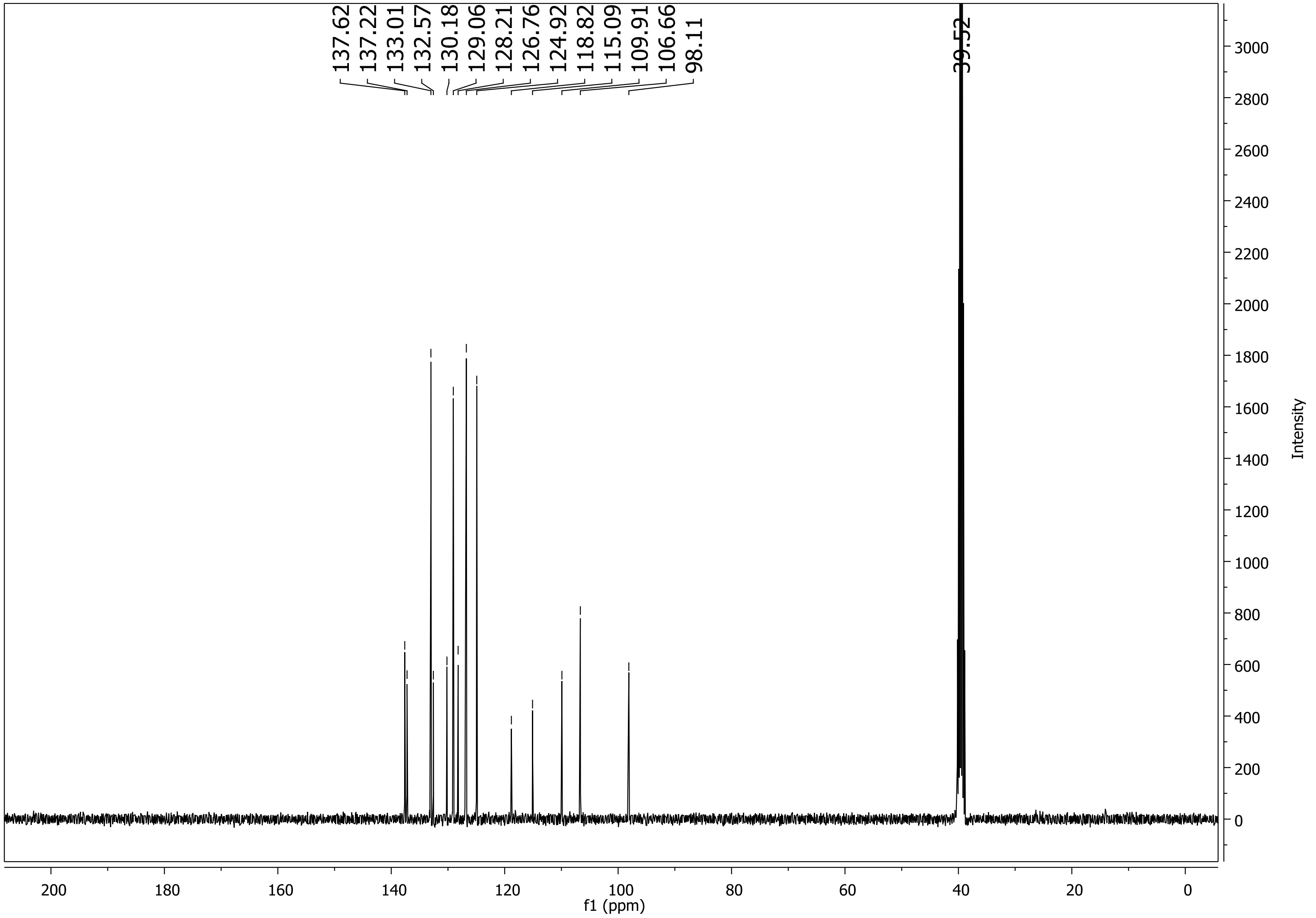

^{13}C NMR (101 MHz, DMSO-d_6) spectrum of **10g**

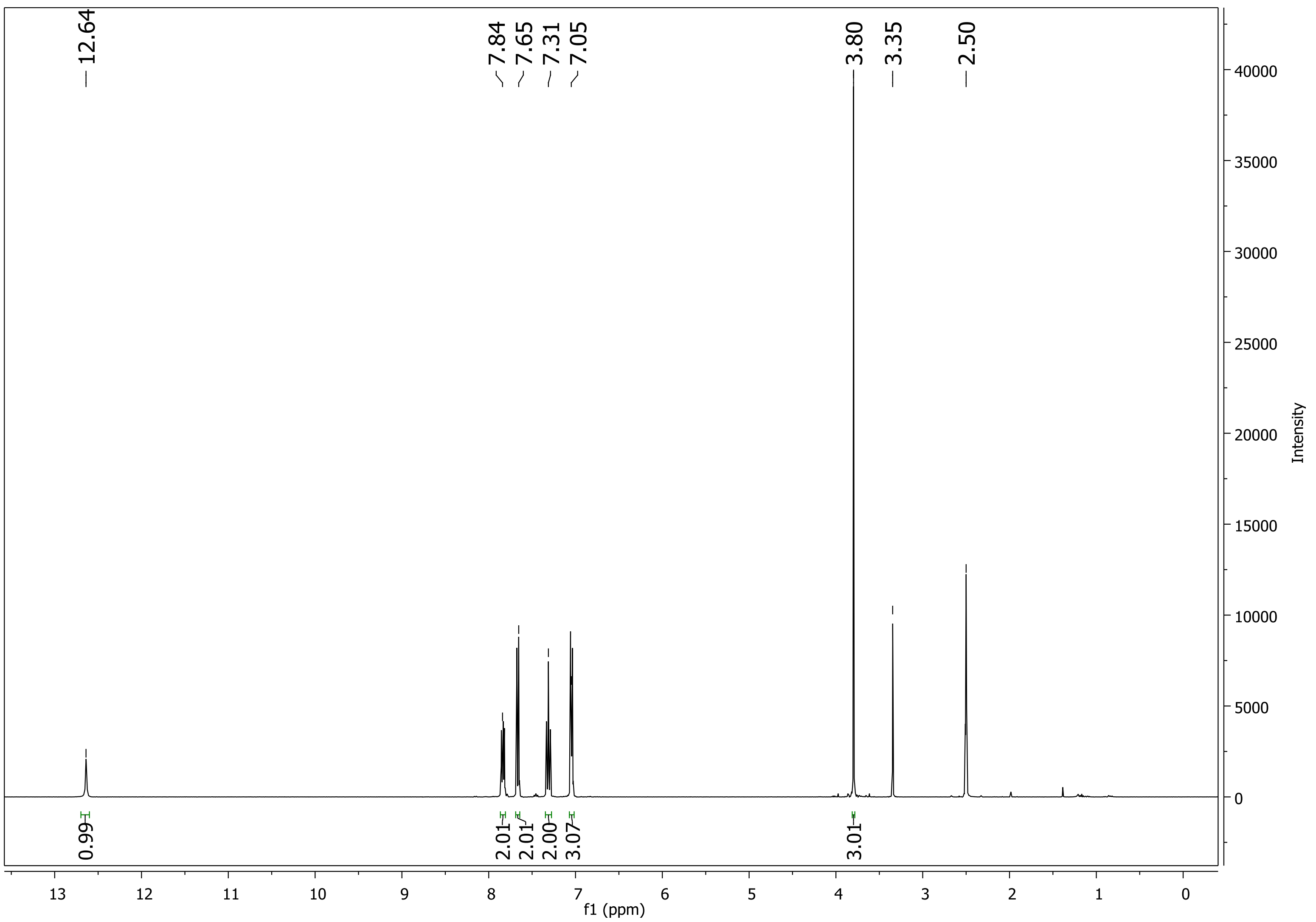

^{1}H NMR (400 MHz, DMSO-d_6) spectrum of **10h**

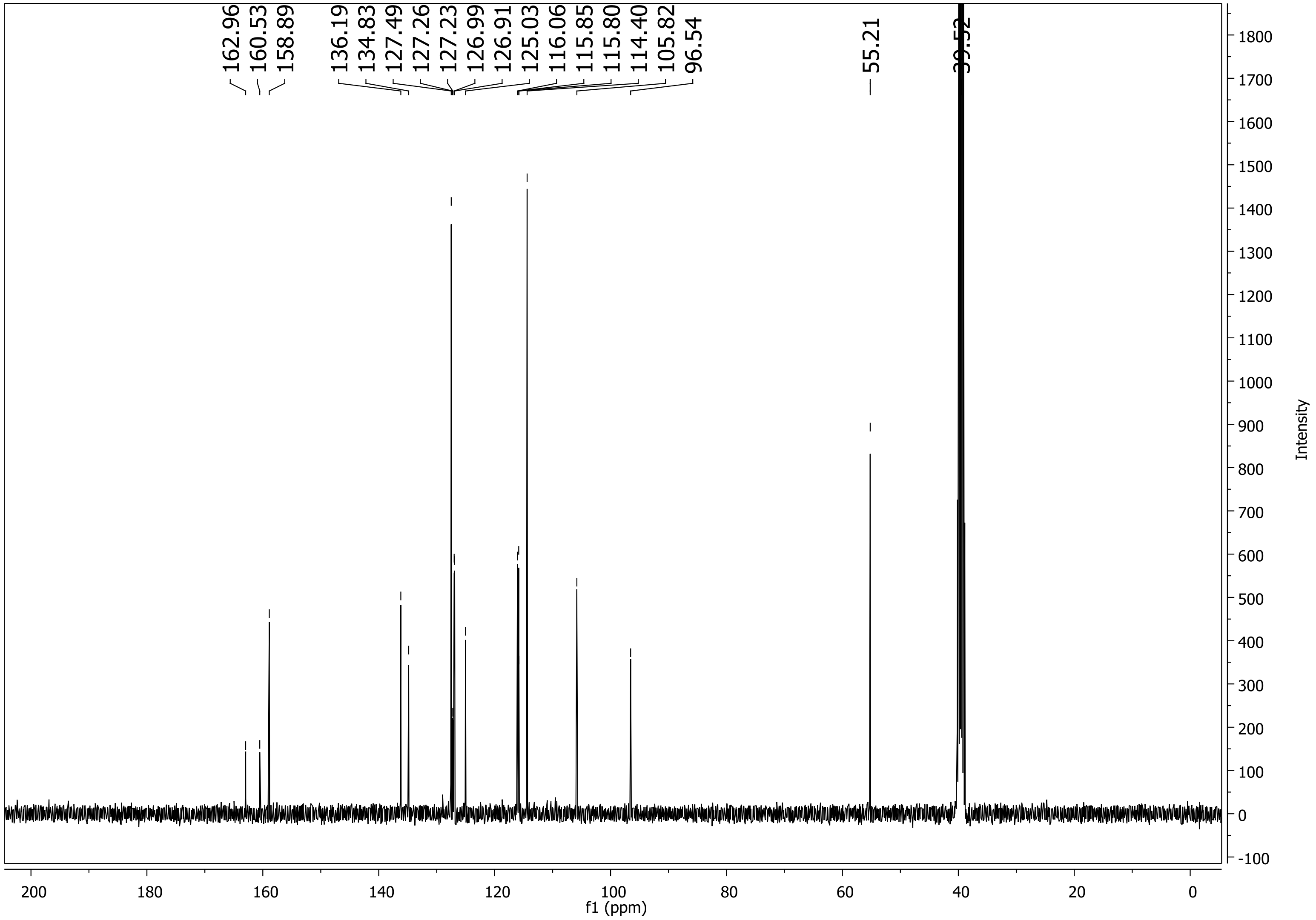

^{13}C NMR (101 MHz, DMSO-d_6) spectrum of **10h**

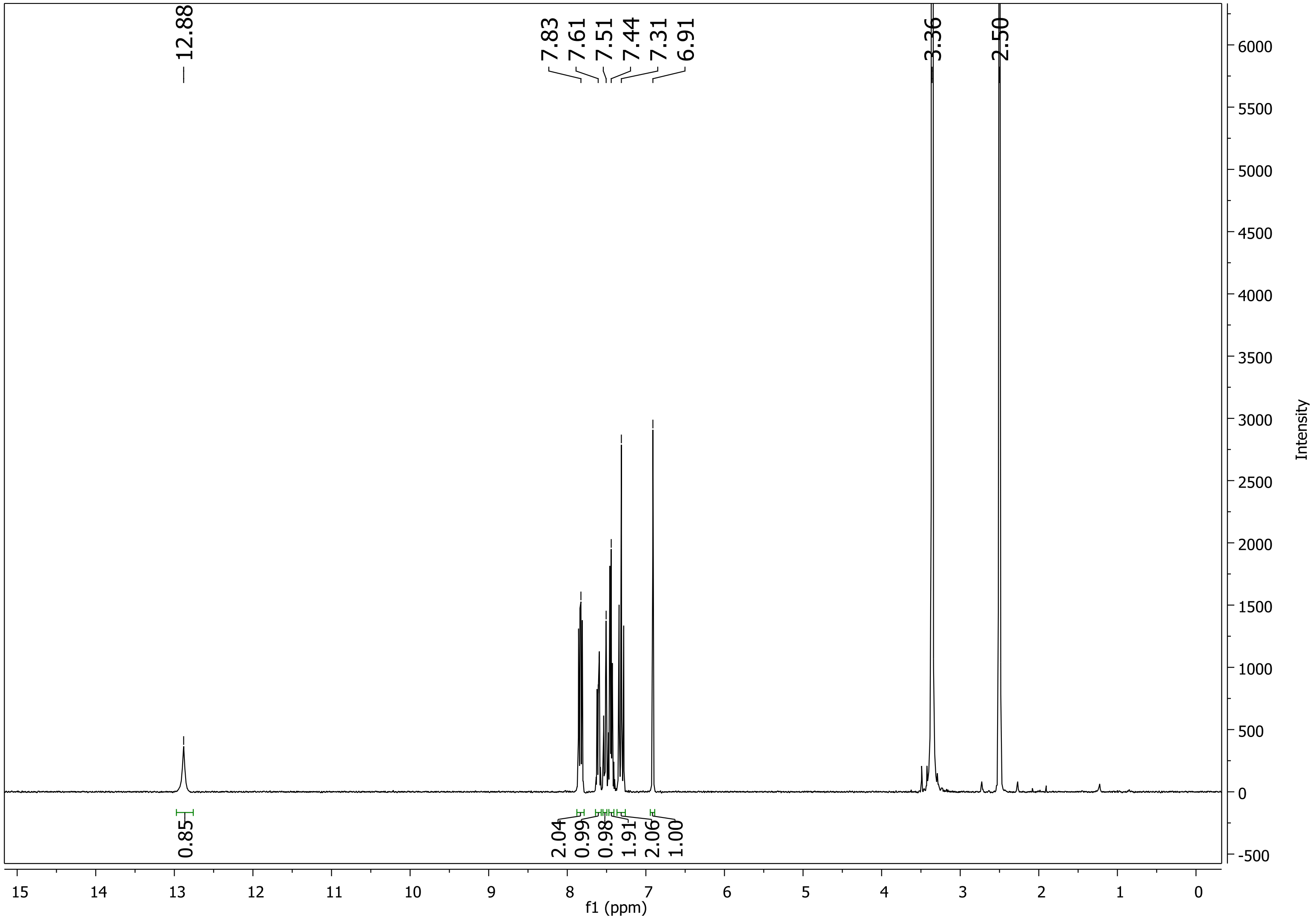

^{1}H NMR (300 MHz, DMSO-d_6) spectrum of **10i**

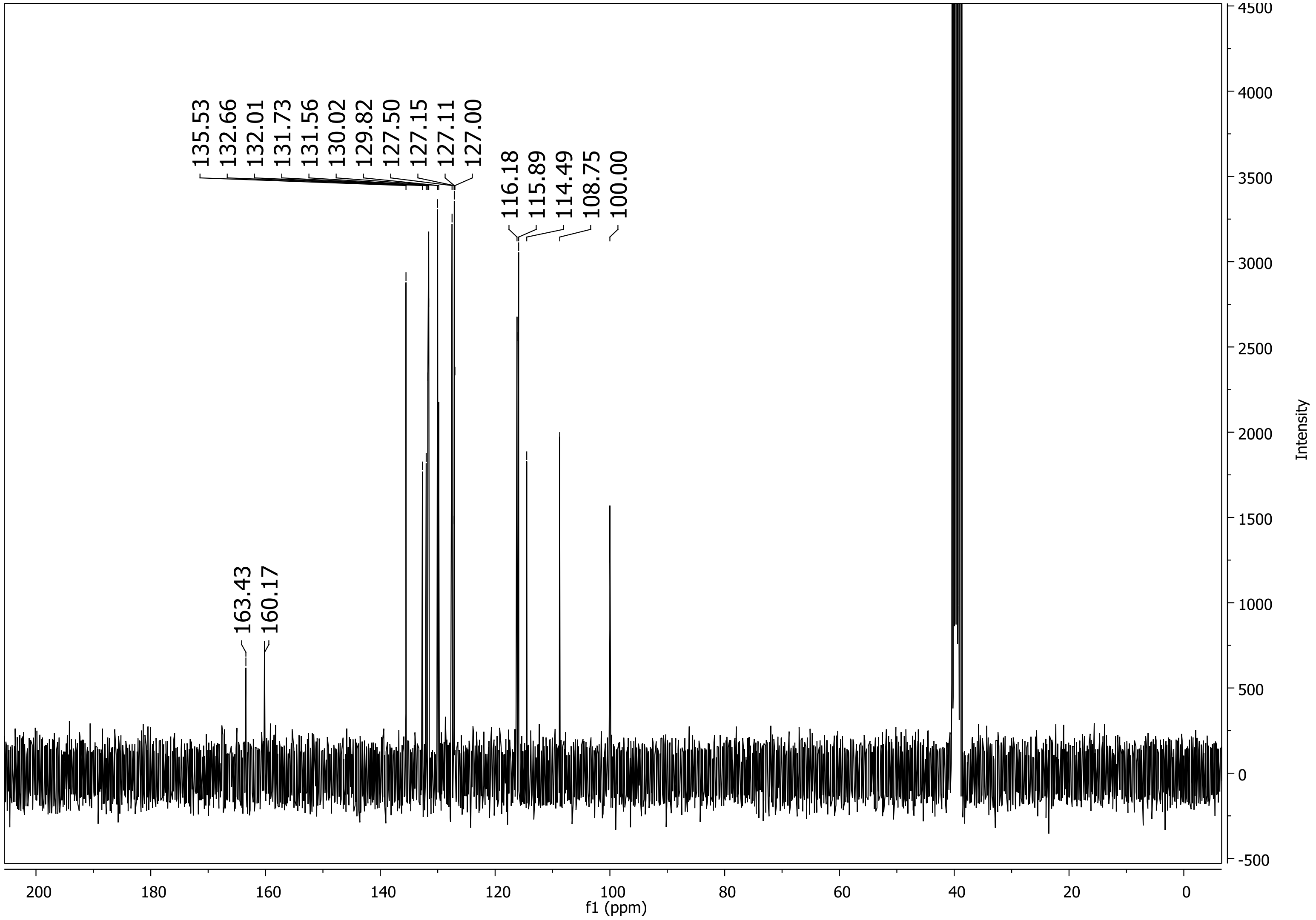

^{13}C NMR (75 MHz, DMSO-d_6) spectrum of **10i**

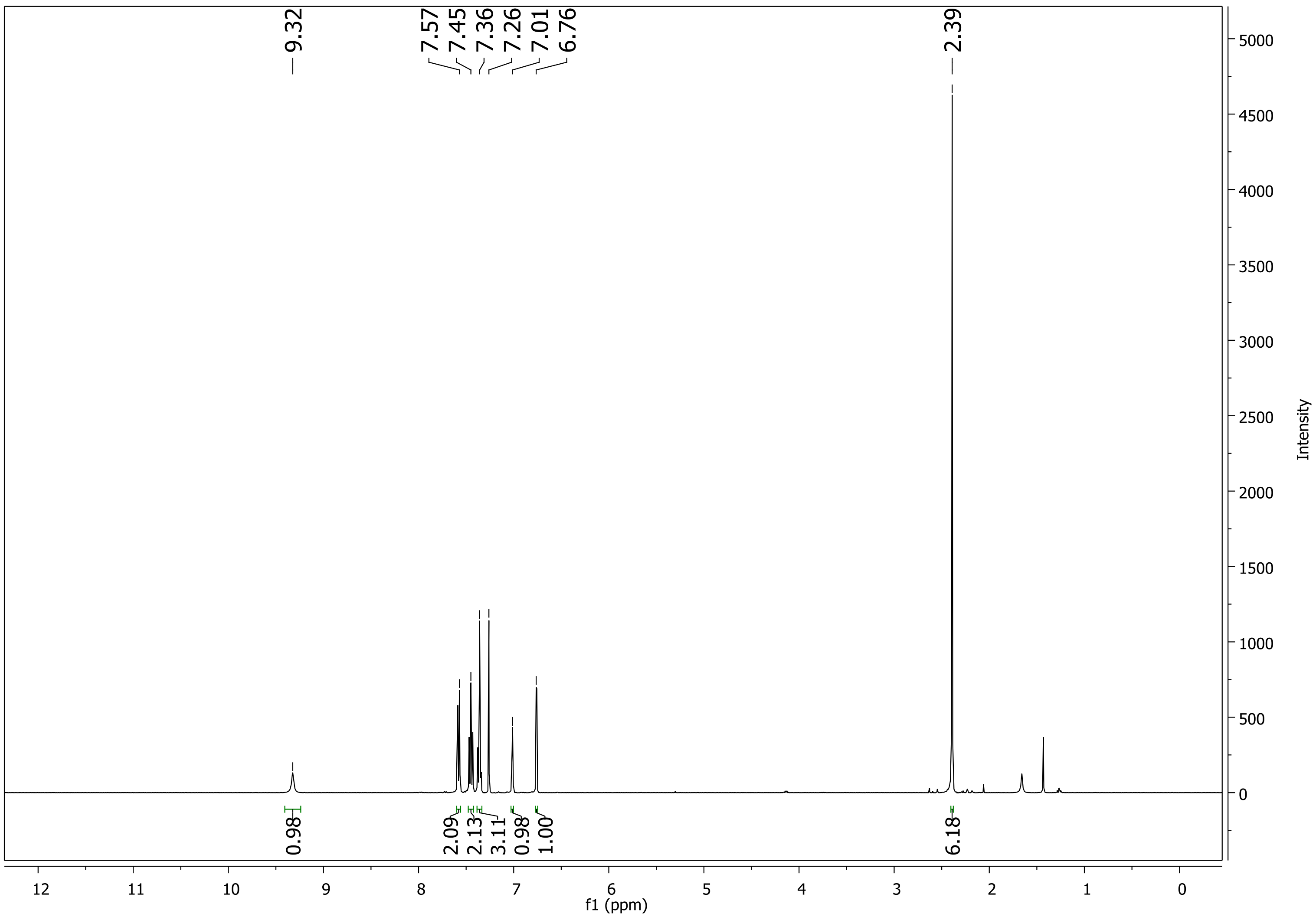

^{1}H NMR (400 MHz, $CDCl_3$) spectrum of **10j**

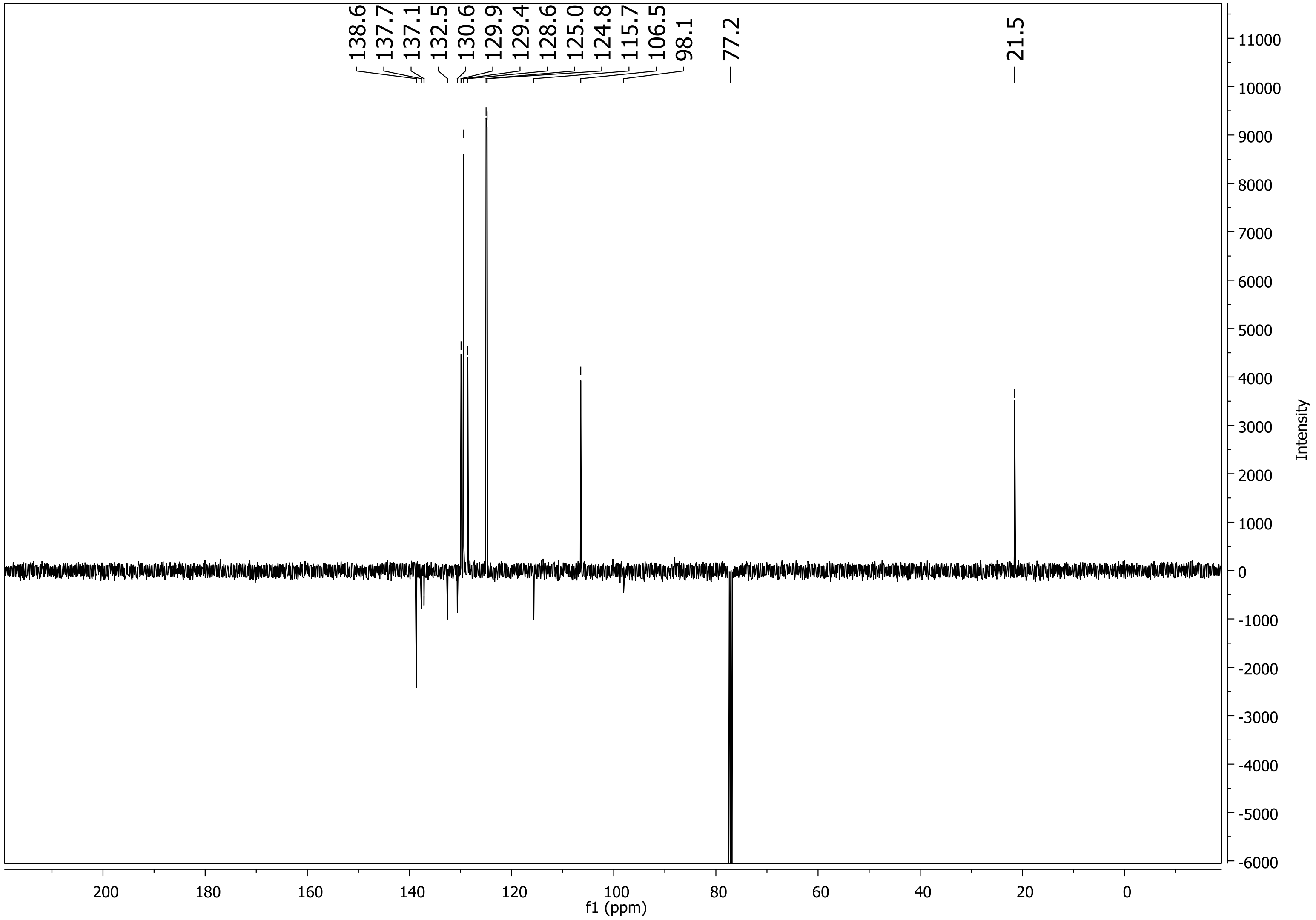

APT NMR (101 MHz, $CDCl_3$) spectrum of **10j**
